# Leguminous Seedborne Pathogens: Seed Health and Sustainable Crop Management

**DOI:** 10.3390/plants12102040

**Published:** 2023-05-19

**Authors:** Eliana Dell’Olmo, Antonio Tiberini, Loredana Sigillo

**Affiliations:** 1 Council for Agricultural Research and Economics, Research Center for Vegetable and Ornamental Crops (CREA-OF), Via Cavalleggeri 25, 84098 Pontecagnano Faiano, Italy; 2 Council for Agricultural Research and Economics, Research Center for Plant Protection and Certification (CREA-DC), Via C. G. Bertero, 22, 00156 Rome, Italy

**Keywords:** legumes, seedborne pathogens, diagnostics, crop management, phytopathology

## Abstract

Pulses have gained popularity over the past few decades due to their use as a source of protein in food and their favorable impact on soil fertility. Despite being essential to modern agriculture, these species face a number of challenges, such as agronomic crop management and threats from plant seed pathogens. This review’s goal is to gather information on the distribution, symptomatology, biology, and host range of seedborne pathogens. Important diagnostic techniques are also discussed as a part of a successful process of seed health certification. Additionally, strategies for sustainable control are provided. Altogether, the data collected are suggested as basic criteria to set up a conscious laboratory approach.

## 1. Introduction

**Figure 1:**
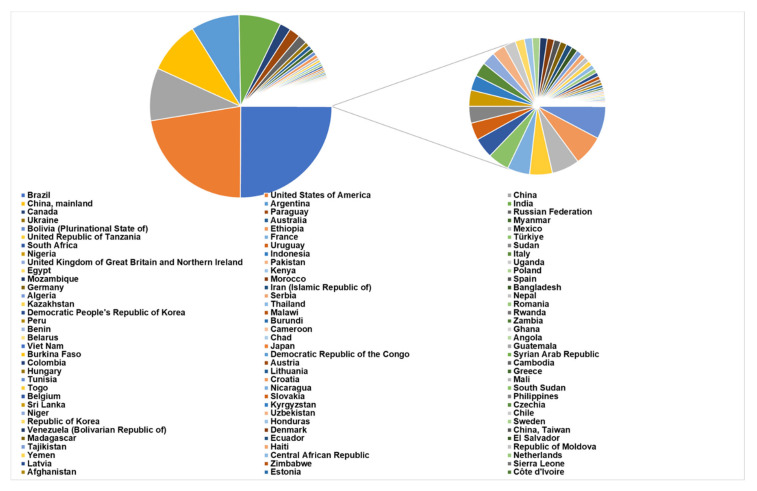
Production (tons) of legumes in different countries across the globe.

In 2021, 551,125,349.85 tons of legumes were produced globally (Figure 1). Brazil is the nation registering the largest value (137,909,415.00 tons), followed by the United States of America (123,382,179.53 tons), China (102,749,390.73 tons), Argentina (47,375,791.63 tons), India (41,289,760.61 tons), and Canada and Paraguay (about 10,000,000 tons each). The total production of legumes in the European Union is 9,367,208 tons, with Italy being the top producer (1,421,600.00 tons). Among legumes, soybean is the most cultivated, with a production of 388,097,786.67 tons, followed by pea (45,863,817.39 tons) and common bean (29,020,607.87 tons) (FAOSTAT https://www.fao.org/faostat/en/#data/QCL, accessed on 5 October 2022).

**Figure 2:**
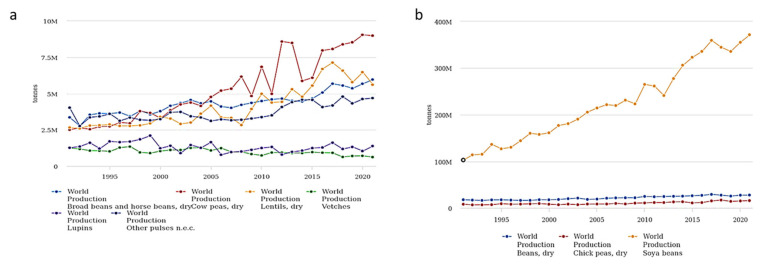
Trends in production of different legumes in the last 25 years. In panel (**a**), the production trends for broad beans, lupins, cowpeas, lentils, and vetches are shown; in panel (**b**), the trends for beans, chickpeas, and soybeans are reported [6].

The Rural Development Policy 2014–2020 encourages the use of legumes in new agricultural systems, because they boost soil fertility by contributing to the recovery of natural organic matter. It is well known that legumes secrete organic acids from the roots, which enables them to solubilize phosphate and, when grown in rotation with other crops, to induce pathogen suppression. Legumes also fix nitrogen (fixation), which allows the enrichment of nitrogen content in the soil [1,2]. Additionally, legumes are recognized as a suitable source of amino acids, proteins, vitamins, and minerals for human consumption [3]. At a global level, the plant-based protein market size is projected to grow from USD 12.2 billion in 2022 to 17.4 billion by 2027. The increased popularity of veganism and flexitarianism as a result of rising consumer awareness of sustainable food production and healthy nutrition, combined with the growth of the global population, enhances the expected income for legumes [4]. Plant proteins are an alternative to meat, as they reduce the risk of obesity and cholesterol and provide micro-elements, minerals, and vitamins to the diet [5]. The interest in legume cultivation is reflected in the growth in production from 1991 to 2021, as shown in Figure 2: in thirty years, the production of dry pea and chickpea has doubled, while soybean increased from 103 million tons to more than 371 million tons. Among minor species, cowpea appears as the crop that mainly increased in production, from 2.5 million tons in 1991 to 9 million in 2021 (Figure 2a,b).

In this scenario, legumes assume a major role in food production. In order to obtain high production, challenges in crop management have to be faced, and research needs to be conducted to increase yields by means of preventing pest spread and devastating diseases. In fact, even though legumes are promising for future food production, current trends have been more focused on the optimization of cereal and oilseed cultivation, leading to the marginalization of Leguminosae crops [7]. Because climatically suited high-yielding pulse cultivars are unavailable, supply networks are not appropriately developed, and agronomic knowledge to assist production is scarce [8]. Moreover, plant species belonging to Leguminosae are vulnerable to a range of seedborne disease, and since they are planted by direct sowing, they are among the plants in which the occurrence of these diseases is a real risk.

In this context, the use of seeds free from pathogens represents the first-line approach to significantly reduce pest dissemination and sheds light on the crucial role played by seed certification in ensuring the health of future cultivation. This review’s goal is to provide a summarized source of pest species that can infect seeds and that should be checked for when aiming to carry out a complete diagnostic screening. Moreover, knowledge of their biology, distribution, host range, and symptomatology are disclosed to provide basic criteria to select the analytical approach. Finally, the main innovative strategies of disease control, with a vision of organic or integrated pest management, are cited to provide elements that are useful to acquire a complete picture of phytosanitary concerns. *Fusarium* spp. are not included in this review, since it is a complex genus that is extensively described in several commendable reviews [9,10,11].

## 2. Seedborne Fungi and Oomycetes of Principal Leguminous Crops

Seedborne fungi directly or indirectly affect seeds by causing seed rot and/or discoloration, as well as by causing seedling diseases that compromise crop yields. Over the years, many pathogens have been associated with pulses, and, in some cases, serious infections and major economic losses have been reported.

### 2.1. Anthracnose (Colletotrichum spp.)

Anthracnose is caused by *Colletotrichum* spp. in a number of plant species, including herbaceous, woody, and cereal plants [12,13] (Table 1). Among *Colletotrichum* spp. hosts, the *Fabaceae* family is the most prevalent. Numerous *Colletotrichum* species have been reported: some, such as *C. nymphaeae* and *C. fioriniae*, have a wide host range, whereas others, such as *C. lupini* and *C. lindemuthianum*, are host-specific [14].

**Figure 3:**
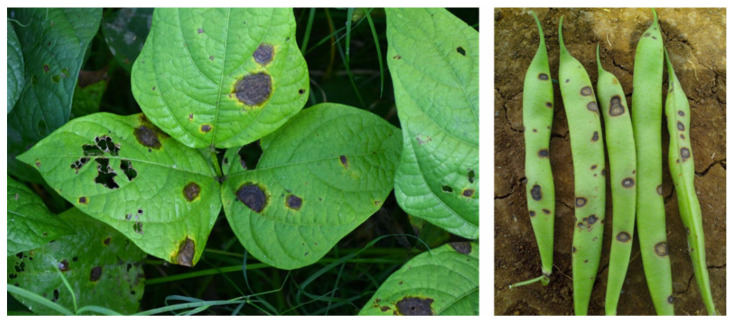
Symptoms of *C. lindemuthianum* on common bean. The images report the typical anthracnose lesions on the leaves (**left panel**) and pods (**right panel**). Photos released by Raja Jeyaraman via Flickr and Gerald Gathogo in PlantwisePlus Knowledge Bank, CABI Digital library [34].

The pathogen *C. lindemuthianum* (Sacc. & Magnus) (1889) belongs to Ascomycota, Pezizomycotina, Sordariomycetes, Hypocreomycetidae, order Glomerellales, family *Glomerellaceae*, and genus *Colletotrichum*, as described in the Index Fungorum database [15]. It overwinters in crop residues or in the seeds, preserved in the seed coat and, more rarely, in the embryo [16]. *C. lindemuthianum* causes disease on the aerial parts of common bean (*Phaseolus vulgaris* L.) plants, and deep and shrunken lesions containing flesh-colored spores are typical symptoms of infection (Figure 3). Usually, lesions are also visible on the hypocotyl, stem, and leaf veins. When the disease progresses, the leaves become chlorotic, then wilt, and, in the final stage, flog. Bean pods can also exhibit symptoms, manifesting as rust-colored lesions that develop into sunken cankers with black ring borders. The disease develops by causing premature pods to die early or the development of dark cankers that make mature pods unsuitable for the market [17]. The pathogenicity of *C*. *lindemuthianum* has been extensively studied, and the evidence implies that conidia germinate on the surface of the host, generating a peculiar melanized germination tube, known as the appressorium. This exerts high turgor pressure on the host surface and penetrates. Once the fungus has entered the host, an infection peg emerges from the appressorium by forming vesicles and primary hyphae (biotrophic). The biotrophic hyphae spread to other cells, and the fungus switches to the necrotrophic phase (secondary hyphae) [18]. Bean anthracnose is one of the most concerning diseases for plant productivity [19], and it can result in 100% yield losses in cool and humid environments [20].

Similarly, *Colletotrichum lupini* (Bondar) Damm, P.F. Cannon & Crous was identified as the causal agent of anthracnose in several lupin species, including blue (*Lupinus angustifolius* L.), white (*Lupinus albus* L.), Andean (*Lupinus mutabilis* Sweet.), yellow (*Lupinus luteus* L.), and ornamental lupins (*Lupinus polyphyllus* Lindl.) [21,22]; the twisting of stems and necrotic lesions on the stems and pods are typical symptoms caused by this fungus. Among legumes affected by *Colletotrichum*, the infection of pea (*Pisum sativum* L.) is also reported. In this crop, anthracnose is caused by *C. pisi* Pat., which is able to produce symptoms on the aerial parts of the plant [23]. Additionally, *C. truncatum* (Schwein.) Andrus & W.D. Moore has been recorded on lentil (*Lens culinaris* Medik.), causing anthracnose as lesions on the leaves, stem, and pods [24]. However, *C. truncatum* is more frequently associated with anthracnose in soybean (*Glycine max* L.), where it can infect plants in all physiological stages [25]. Notably, anthracnose in soybean causes pre- and post-emergence damping off and dark and depressed spots on the leaves, stem, and petioles that finally lead to the early defoliation of the plant [26]. Furthermore, as reported for other species, anthracnose in soybean can result in 100% yield losses [27]. In addition to the well-known hosts for *Colletotrichum* spp., in 2022, a case of anthracnose caused by *C*. *cliviicola* Damm & Crous was documented in Nigeria in cowpea (*Vigna unguiculata* L.). The symptoms were reddish-brown spots, necrotic lesions, and vein streaks [28]. In the same year, a report from Thailand revealed anthracnose in butterfly pea (*Centrosema pubescens* Benth.), which manifested as necrotic spots on leaves with a chlorotic halo [29]. A similar case was described for alfalfa (*Medicago sativa* L.), in which *C. trifolii* Bain was identified as the causal agent of anthracnose; the fungus causes devasting symptoms on the stem and crown worldwide [30]. Finally, further reports of different new species causing anthracnose on alfalfa have been described across the globe: *C. linicola* in Serbia, *C. truncatum* in Turkey, and *C. americae-borealis* in Argentina [31,32,33].

### 2.2. Charcoal Rot (Macrophomina phaseolina)

*M. phaseolina* Tassi (Goid.) is an ascomycete belonging to the family Botryosphaeriaceae [35,36], which includes pathogenic, necrotrophic, and endophytic fungi [37]. This fungus is present all over the globe, and it is one of the major pathogens for legumes (Table 1); it has been noted for having a wide host range, including more than 700 plant species [38,39,40]. Taxonomically, the pathogen is classified as Ascomycota, Pezizomycotina, Dothideomycetes, Incertae sedis, order Botryosphaeriales, family *Botryosphaeriaceae*, and genus *Macrophomina* [41].

**Figure 4:**
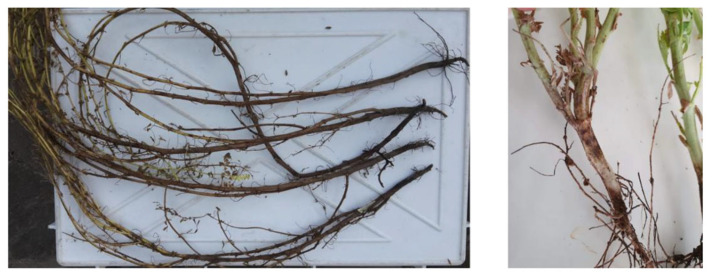
Symptoms of *M. phaseolina* charcoal rot in chickpea plants published in Dell’Olmo et al., 2022 [40].

*M. phaseolina* is able to act as both a soilborne and seedborne pathogen; it colonizes seeds mostly under the seed coat and induces seed deterioration and germination failure [42], causing significant losses in crops [43]. Usually, *M. phaseolina* is preserved as microsclerotia that can survive up to 15 years and represents the primary source of inocula, both in the soil and embedded in the plant tissues [44,45]. The infection process caused by *M. phaseolina* can be divided into four stages: (i) germination, in which the microsclerotia germinate into hyphae; (ii) penetration, in which the formed appressoria allow *M. phaseolina* to penetrate the host cells; (iii) parasitic phase, the stage in which the pathogen colonizes host cells and disrupts nutrient and water transport; (iv) saprophytic phase, the expression of vascular disease that leads to plant wilting and permanent decay [36,44,45]. Symptoms linked to *M. phaseolina* infection can be various, since the pathogen can cause crown charcoal rot, root rot, and stem rot (Figure 4) [46]. Infections with *M. phaseolina* have been reported for several legumes, including common bean, cowpea, chickpea, pea, soybean, lentil, and alfalfa [17,40,45,47,48,49]. Interestingly, in 2021, a report of charcoal rot in lentil caused by *M. pseudophaseolina* Crous, Sarr & Ndiaye was described for the first time worldwide [50]. Several studies have been carried out to find correlations among *M. phaseolina* isolates based on the pathogenicity, host, or geographical origin, but, in most cases, the results have been found to be fruitless [40,51]. Additionally, the results of these studies evidenced the high genetic variability typical of this species.

### 2.3. Ascochyta Blight (Ascochyta spp. and Phoma spp.)

**Figure 5:**
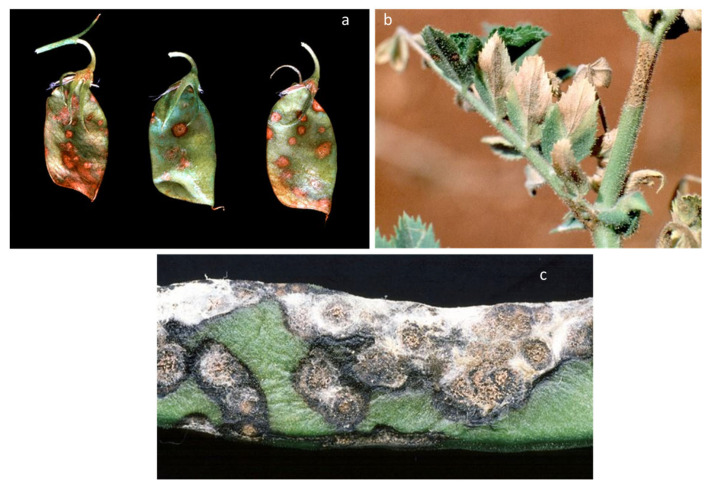
Ascochyta blight symptoms on (**a**) lentil, (**b**) chickpea, and (**c**) faba bean. Photos released by Robin A.A. Morrall and M. Fawaz Azmeh, published in CABI compendium 2022, CABI Digital library [69,70,71].

Ascochyta blight represents one of the major threats of cool seasoning grain legumes (Table 1), including chickpea, pea, faba bean, and lentils [52]. *Ascochyta* is taxonomically classified into the phylum Ascomycota, class Dothideomycetes, subclass Coelomycetes, order Pleosporales, family *Didymellaceae*, and genus *Ascochyta* [41]. Usually, the pathogen is host-specific, causing disease in only one crop: for instance, *Ascochyta rabiei* (Pass.) Labr. is found in chickpea, *Ascochyta pisi* Lib. is found in pea, *Ascochyta fabae* Speg. is found in faba bean, and *Ascochyta lentis* Vassiljevsky is found in lentil [53]. The genus *Ascochyta* includes only necrotrophic pathogens that are able to kill the host cells in advance of mycelial development due to the production of toxins and lytic enzymes [52]. It has been proved that *A. rabiei* is stored in the soil, on or inside the seeds, and on plant debris in various forms, such as mycelia and pycnidia. Previous studies proved that both *A. rabiei* and *A. pisi* [54,55] can be found in the seed coat and, at lower concentrations, in the embryos [56]. The same strategies of storage can be extended from *A. rabiei* to other *Ascochyta* species, such as *A. lentis* and *A. pisi* [57,58]. Pathogen spread can occur by seed transmission, which was the cause of the documented introduction of *A. rabiei* in Canada, Iran, and Australia; otherwise, over short distances, the spores can diffuse in the environment through air movements [58]. The infection starts when climatic conditions are favorable, such as a temperature ranging from 15 to 30 °C and high moisture. In the initial stage, *Ascochyta* spores germinate on the leaves and appressoria develop. The mechanical pressure allows pathogen penetration within epidermic cells, and the mycelium grows parallelly between the epidermal and parenchyma cells, causing the disrupting of the inner leaf structure. On the other hand, histological studies proved that the host cells were disintegrated even before direct contact with the fungus, suggesting the involvement of phytotoxic compounds causing the necrosis [59]. In chickpea, seedborne *A. rabiei* infection causes brown lesions at the stem base that worsen with time until the stem breaks and the plant dies (Figure 5) [56]. In contrast, in the field, the first symptoms are the appearance of concentric necrotic lesions on all aerial parts of the plant, and, in favorable conditions, 100% of the plants can die [60]. In pea, infections with *A. pisi* on seeds decrease the germination rate and seedling vigor [57,61]; symptoms on adult plants range from irregular purplish-brown/black spots on leaves in minor infections to stem and pod lesions in more severe cases [62]. Similarly, in lentil and faba bean, *A. lentis* and *A. fabae* produce circular dark-brown spots on leaves [58,63]. Symptoms of Ascochyta leaf blight have also been described in common bean and cowpea, where the causal agents were identified as *Phoma exigua* var. *exigua* and *Ascochyta phaseolorum*, respectively. Furthermore, in both common bean and cowpea, the economic losses caused by the disease are huge [64,65]. Regarding soybean, the first description of Ascochyta infection was reported in 1931, and the pathogen was identified as *Ascochyta sojicola*, now named *Phoma sojicola* (Abramov) Kövics, Gruyter & Aa [66]. In this host, the infection can start from the seeds, involving cotyledons that show necrotic spots [66]. The seedlings then die after two weeks or, if they survive and are able to grow, develop v-shaped lesions on their leaves [66]. Additionally, studies performed on alfalfa seedlings, in which mortality reached up to 90%, revealed a high incidence of *Ascochyta imperfecta* Peck. in seeds [67]. In 2007, infections caused by *A. rabiei* in *Vicia hirsuta* L. were reported in Georgia, with symptoms similar to those usually observed in chickpea [68]. Then, in 2016, Ascochyta blight was reported in Canada on Fenugreek (*Trigonella foenum-graecum*) and was caused by infection with *Peyronellaea pinodes* (preferred name: *Dydimella pinodes*).

### 2.4. Root Rot and Seed Rot (Rhizoctonia solani)

**Figure 6:**
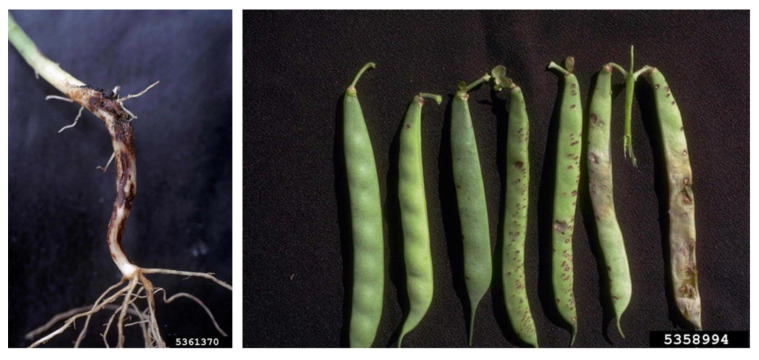
Symptoms of *R. solani* on the collar and pods of dry beans. Photos licensed under a Creative Commons Attribution 3.0 License, supplied by Howard F. Schwartz, Colorado State University, Bugwood.org [95].

*Rhizoctonia solani* J.G. Kühn is one of the most widespread soilborne pathogens that inflict massive damage to economically important crops worldwide [72]. It has been classified as Eukarya, kingdom Fungi, subkingdom Dikarya, phylum Basidiomycota, subphylum Agaricomycotina, class Agaricomycetes, order Cantharellales, family *Ceratobasidiaceae*, and genus *Rhizoctonia* [41]. The symptoms associated with *R. solani* (Table 1) infection are various and change based on the host: they include hypocotyl, crown, stem and root rot, blights, wire stem, and damping off [72]. Being a seedborne pathogen, it can lead to seed rot and decay, pre-emergence damping off, and germination failure [73,74]. Moreover, it has been demonstrated that *R. solani* can be preserved in different parts of the seeds, including the seed coat, endosperm, and embryos [75]. *R. solani* is usually preserved in the soil or plant debris as sclerotia or, in seeds, in the form of mycelium. Otherwise, the teleomorph form permits the propagation of basidiospores that are carried by the wind and rain, but this form is rarely encountered in fields [74]. *R. solani* overwinters in the soil, and the infection starts with mycelium germination through the plant epidermis. At this stage, leguminous plants produce an exudate that drives the response of *R. solani*, which grafts a hyphal attachment to the host surface, with the consequent formation of a T-shaped appressorium-like structure [76]. Then, *R. solani* can penetrate the host epidermis and grow along the cell walls of the host cells. From then on, *Rhizoctonia* grows in the root cortex, leading to progressive colonization inside the root and on the root surface. In favorable conditions, the fungus generates dark streaks that can result in root rot and root death [74]. Evidently, the dramatic symptomatology of the roots also has consequences on the aerial parts of the host, where typical symptoms of *R. solani* consist of chlorosis, blights, stunting, and leaf wilting [74]. The disease cycle terminates when the sclerotia, stored in the infected seeds or in plant wastes, return to the soil, where they survive for a long time [77]. Interestingly, *R. solani* has been described as a complex, and as a result, it has been divided into 15 somatic-incompatible groups, also known as anastomosis groups (AGs), taking into account hyphal fusion, morphological diversity, differences in biochemical and molecular markers, and diverse pathogenicity and aggressiveness [72,76,78,79]. Therefore, AGs are differentially represented on the global scale: indeed, AG1, AG2, AG3, and AG4 are the most widespread, while others are specific to certain regions (AG-5 has been found in Canada, Germany, Israel, and Japan; AG-7 has been found in Japan; AG-8 has been found in Australia; AG-9 has been found in America and Canada; and AG-BI has been found in Japan). Among these, the AG5 group is the most commonly found in legume crops [80,81], such as common bean, pea, chickpea, faba bean, and soybean. In common bean, *R. solani* can affect plants at any stage of development. It can be either soilborne or seedborne, and, in the latter case, the pathogen can compromise the seed yield by up to 100% [78,82]. Typical symptoms are root rot, but, in some cases, the infection can also lead to the defoliation of the aerial parts [83], the damping off of early seedlings, or stem and root rot in the oldest plants [84]. Similarly, *R. solani* has been found to be the main pathogen responsible for root rot in chickpea (*Cicer arietinum* L.) worldwide, and, during a study performed in India, it has also been recognized as the causal agent of wet root rot on this crop [74,85,86], confirming *R. solani* as one of the key pathogens in chickpea cultivation [87]. As mentioned before, the necrotrophic nature of *R. solani* allows the infection of a wide host range, which also includes pea, causing seedling blights, damping off, and root rot, and lentil, in which it induces seedling damping off and, under severe pathogen pressure [88,89,90], the total loss of the harvested crop [74]. *R. solani* also exhibits seedborne behavior in faba bean, in which, in different global regions, it has been reported as responsible for root rot, collar rot, defoliation (Figure 6) [91,92], and the frequent mortality of plants. In the past, massive economic damages caused by the fungus on this crop forced farmers to abandon their lands, with consequent economic losses also aggravated by the decrease in the plot value [93]. Among *R. solani* hosts, soybean also has to be mentioned. This pathogen is able to attack both the aerial and underground parts of soybean plants, and it was demonstrated that the outcomes of the disease are strictly dependent on the phenological phase at the stage of infection. In the case of seedborne disease, replanting in the field can be required due to seedling decay, damping off, and the final death of the plants. A similar scenario can be observed when the pathogen reaches old plants that are not able to react to the pathogen invasion, resulting in a yield loss of up to 48%. On the other hand, young soybean plants are able to withstand infections by *R. solani*, to stand, and, in some cases, to continue growing [94]. Finally, *R. solani* is also among the most widespread diseases of alfalfa, causing the premature death of seedlings [76].

### 2.5. Diaporthe/Phomopsis Complex

**Figure 7:**
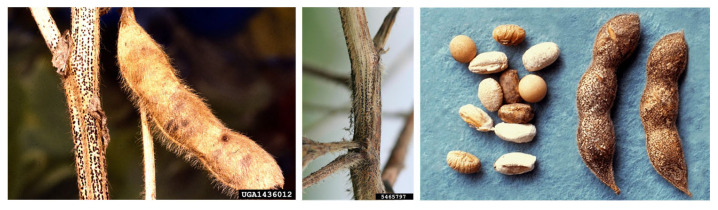
Symptoms of *D. phaseolorum* var. *sojae* and *Phomopsis sojae* on soybean stem, pods, and seeds. Photos supplied by Clemson University—USDA cooperative extension slide series/via Bugwood.org—CC BY 3.0 and Daren Mueller, Iowa State University, bugwood.org, published in PlantwisePlus Knowledge Bank, CABI Digital library [105].

The genus *Diaporthe*, together with its anamorph *Phomopsis*, consists of a huge number of species, and it includes phytopathogenic, saprophytic, and symbiotic endophytic fungi [96,97]. Taxonomically, it has been classified as Eukarya, kingdom Fungi, subkingdom Dikarya, phylum Ascomycota, class Sordariomycetes, order Diaporthales, family *Diaporthaceae*, and genus *Diaporthe* [41]. Numerous important horticultural crops are among the vast variety of hosts that *Diaporthe/Phomopsis* spp. can infect (Table 1), leading to significant production reduction and financial losses [98]. *Diaporthe* is recognized as a key pathogen of soybean, acting primarily as a seedborne agent, and is reported to be able to colonize the seed coat rather than the embryos [99]. In soybean, *Diaporthe* species are the causal agents of seed decay, seed rot, pod and stem blights, and stem cankers (Figure 7) [98]. However, pathogens belonging to this genus are able to infect several plant species, producing a variety of symptoms, including damping off, leaf spots, dieback, wilt, and fruit and root rot [97,100,101]. Usually, soybean seeds colonized by *Diaporthe/Phomopsis* are smaller than healthy ones, but they do not show other evident symptoms. Otherwise, in some cases, the seeds can be broken and covered with grayish-white mycelium [97,102]. Extensive taxonomic studies have been performed, and initially, the colony morphology, the host of the infection, physiology, and aggressiveness were utilized to identify the components of the *Diaporthe* genus [97]. Since then, due to the great variability that characterized this genus, the above-mentioned parameters have been considered unsatisfactory for classification, and thus, more accurate molecular analyses have been performed. A study carried out on *Diaporthe* isolates collected in Europe showed that *Diaporthe longicolla* (Hobbs) J.M. Santos, Vrandecic & A.J.L. Phillips was the most represented, followed by *Diaporthe eres* Nitschke, *Diaporthe novem* J.M. Santos, Vrandečić & A.J.L. Phillips, and *Diaporthe caulivora* (Athow & Caldwell) J.M. Santos, Vrandecic & A.J.L. Phillips [103]. Similar studies have been conducted in North Dakota, where *D. longicolla* was isolated from reddish-brown lesions on the stems of dry pea, dry bean, and soybean [104]. Furthermore, in 2015, *Diaporthe* strains isolated from the leaves of common bean collected in Brazil were subjected to a multi-locus phylogenetic analysis that revealed the prevalence of *D. infecunda* R.R. Gomes, Glienke & Crous, while *D. phaseolorum* (Cooke & Ellis) Sacc. was found only in two samples. Another species of *Diaporthe*, namely, *Diaporthe toxica* P.M. Will., Highet, W. Gams & Sivasith., is associated with narrow-leafed lupin (*Lupinus angustifolius* L.), and it is responsible for lupinosis, a mammalian life-threatening disease caused by mycotoxins. The gravity of *Diaporthe/Phomopsis* disease is also due to the endophytic behavior of this pathogen, which develops lesions on the stem only after a long steady phase when optimal conditions occur. The *Diaporthe/Phomopsis* complex is devastating at the seed level, and the control of seed health is pivotal to counteract the disease and its dispersion.

### 2.6. Gray Mold (Botrytis spp.)

*Botrytis* is an Ascomycota, taxonomically classified in the kingdom Fungi, phylum Ascomycota, subphylum Pezizomycotina, class Leotiomycetes, order Helotiales, family *Sclerotiniaceae*, and genus *Botryotinia* [41]. *Botrytis* infection occurs on a wide range of legumes, including chickpea, lentil, faba bean, and pea. Moreover, the pathogen is widely distributed all over the globe, acting as a seedborne and soilborne pathogen and causing extensive economic losses. The conservation organs of this fungus, named sclerotia, are relevant in the *Botrytis* life cycle. They represent the main strategy for the survival of the pathogen, and thus, they are stored in plant debris and, in some species, can reside under the seed coat, serving as an inoculum source. In the spring, sclerotia germinate and start to grow in host tissues, producing conidiophores and multinucleate conidia, which represent the primary source of inocula in the field. Moreover, *Botrytis* spp. can also complete a sexual cycle, in which the spermatization of sclerotia leads to the production of apothecia and asci with eight binucleate ascospores [106]. The pathogenicity of *B. cinerea* Pers. is determined by several environmental factors, including pH, UV radiation, and the percentage of humidity [107]. Infections with B. *cinerea* in chickpea are spread worldwide and generate massive crop losses, especially when optimal conditions occur. The pest symptoms appear near the ground level as water-soaked lesions on the stem, which can extend alongside the tissue. Branches are susceptible to breaking at the rotting points, extending the disease to the leaves and the flowers. The infection can also involve the pods, where, in favorable conditions, the fungus can form gray or brown to light-brown water-soaked lesions [108]. *B. cinerea* and *B. fabae* are identified as the causal agents of gray mold in lentil. Both pathogens have been isolated from the seeds, and, in Australia, it has been reported that seedborne *Botrytis* infections caused 20% of crop losses. Symptoms start in the lower leaves, which finally drop, and lesions appear on the stem as light-brown spots covered by gray mold. The progress of the disease leads to crown rot and dry rot of the entire plant [108].

*B. cinerea* is also able to infect common bean, pea, and soybean. It affects the pods and, as a consequence, decreases the crop yield and the economic crop income. When favorable conditions occurred, crop losses were registered at up to 50%. In common bean, the disease usually begins in senescent flowers and cotyledons; then, the pathogen establishes itself in the pods, where it acts as the initial inoculum, responsible for the subsequent soft rot. Otherwise, when the infection starts on the stem, lesions can progress, leading to plant collapse. In pea, the symptoms of the disease can be visually observed as milky-white coloration on infected seeds; they have a decreased germination level and represent the starter of seedling rot [109]. On soybean, *B. cinerea* can be seedborne, but it is considered a minor disease in this species [108,110,111].

**Figure 8:**
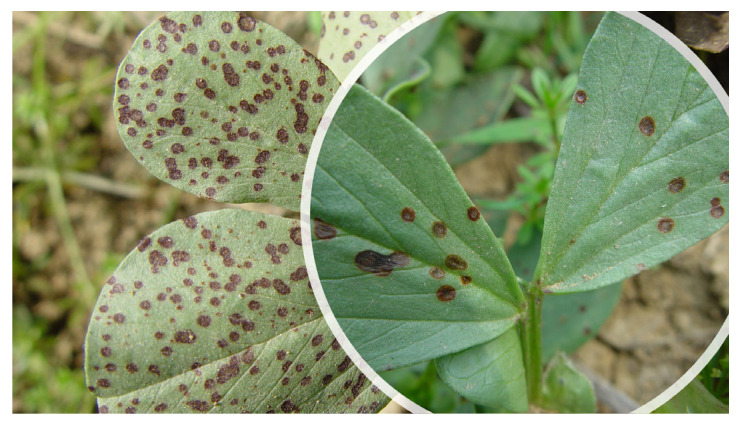
Chocolate spot caused by *B. fabae* on faba bean published in Plantwise Plus Knowledge Bank 2019, CABI Digital library [112].

On the contrary, *B. fabae* Sardiña is the main cause of chocolate spots in faba bean, and symptoms range from small necrotic spots to extensive plant destruction (Figure 8). The infection starts with a non-aggressive phase, manifesting in the appearance of reddish-brown or chocolate-brown spots on all the aerial parts of the plant: leaves, stem, pods, and flowers. During the season, with the increase in humidity, the infection switches to the aggressive phase, in which the lesions expand from 2–3 mm to 5–10 mm in diameter and merge, forming black blight on the leaves and stems. The lesions are irregular in shape, with light and dark concentric ridges that often develop during their expansion. The fungus can also grow saprophytically on fallen dead leaves, sporulate, and re-infect younger, growing leaf tissues. Chocolate spot disease is widespread all over the globe but assumes the most relevance in the most humid regions [108].

### 2.7. White Mold (Sclerotinia sclerotiorum)

**Figure 9:**
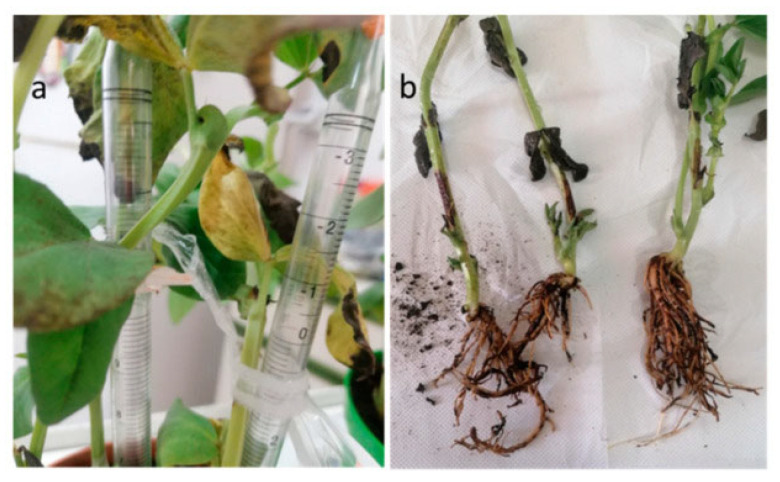
Symptoms of *S. sclerotiorum* on faba bean; (**a**) chlorosis on the leaves and (**b**) root rot. The image was uploaded by Dell’Olmo et al., 2022 [119].

*Sclerotinia sclerotiorum* (Lib.) de Bary is reported as a seedborne pathogen in at least 26 plant species, including legumes. Taxonomically, the pathogen is classified in the kingdom Fungi, phylum Ascomycota, class Discomycetes, order Helotiales, family *Sclerotiniaceae*, and genus *Sclerotinia* [41]. The pathogen produces significant yield losses in many crops across the world, and when it affects the pods, the losses can reach 100% [113]. Furthermore, the disease can involve all parts of the plant, with acropetal development. During the infection, lesions occur on the petioles, branches, and stem, with the final evasion of gray mycelium on all of the organs involved [114]. In the advanced stages of the disease, the pods appear discolored and are characterized by soft rot. *S. sclerotiorum* is preserved as sclerotia that are white in the beginning but that become dark brown or black when they are mature. This form serves as the primary inoculum and can preserve the pathogen in the soil for more than 8 years [115]. Depending on environmental conditions, sclerotia can germinate carpogenically or myceliogenically [116,117], resulting in two different types of diseases. In the former, sclerotia germinate, generating apothecia and ascospores that infect the aerial portions of host plants, while, in the second case, sclerotia produce hyphae that can directly infect the tissues. In legumes, the mycelium develops an appressorium able to directly use enzymes or produce mechanical force to penetrate through the stomata [114]. White mold has been reported as a seedborne pest for common bean and soybean, producing issues in the germination stage. In fact, Nicholson and coworkers demonstrated that seeds affected by *S. sclerotiorum* were flatter, smaller, and discolored, with decreased germination relative to healthy ones [118]. Seedborne infection with *S. sclerotiorum* was also observed in faba bean in Italy in 2022; the experimental infection of plants showed severe symptoms, ranging from leaf chlorosis to stem rot (Figure 9) [119].

### 2.8. Aphanomyces Root Rot (Aphanomyces euteiches)

*Aphanomyces euteiches* W.F. Pfender & D.J. Hagedorn is a major pathogen with a significant economic impact in pea, lentil, and alfalfa. Taxonomically, it has been classified in the kingdom Chromista, phylum oomycote, class Peronosporea, order Saprolegniales, family *Leptolegniaceae*, genus *Aphanomyces,* and species *euteiches* [41]. *A. euteiches* is globally spread, and the symptoms of the disease are similar in all host plant species. At the beginning of the disease, roots appear gray in color and are water-soaked. When the disease worsens, the roots become golden-brown and soft, along with the decay of lateral roots and root hairs. The root system appears reduced in size, and the overall functions are limited. In later disease stages, the symptoms spread upward to the epicotyl, the infection progresses, and chlorosis and necrosis of leaves become visible. In the most severe cases, the stunting and death of the plant may occur. The disease symptoms may be worse when soils are compacted due to compromised root system development [120].

## 3. Seedborne Bacterial Pathogens of Principal Leguminous Crops

### 3.1. Common Bacterial Blight (Xanthomonas spp.)

**Figure 10:**
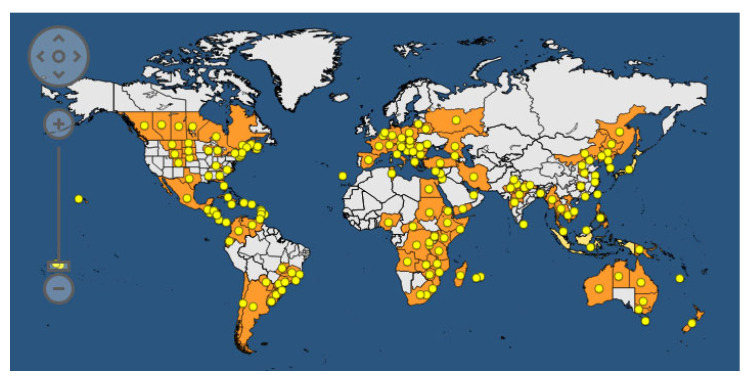
Global distribution of common bacterial blight. EPPO 2023. The countries affected by common bacterial blight are reported in orange; the yellow dots represent the permanent presence of the pathogen in the country.

Common bacterial blight of bean (CBB) is spread all over the world. According to the EPPO (European Plant Protection Organization) Global Database, updated to 2023 [121], the disease is present in 170 countries on all five continents (Figure 10).

**Figure 11:**
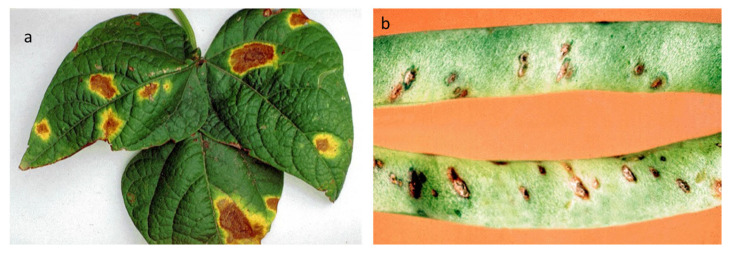
Symptoms of Xpp on common bean (**a**) leaves and (**b**) pod. Photos released by P.H. Goodwin and Mauritius Sugar Industry Research Institute, published in CABI Compendium 2019, CABI Digital library [125].

CBB was recorded for the first time in 1893 and represents one of the most studied seedborne diseases of legumes. The causal agents are *Xanthomonas phaseoli* pv. *phaseoli* (Xpp) (ex. *Xanthomonas axonopodis* pv. *phaseoli*) and *Xanthomonas citri* pv. *fuscans* (Xcf) (ex *X*. *a*. pv. *phaseoli* var. *fuscans*). The EPPO database reports the taxonomic classification of Xpp and Xcf as follows: Bacteria; Proteobacteria; Gammaproteobacteria; Lysobacterales; *Lysobacteraceae*; *Xanthomonas*; *Xanthomonas phaseoli* pv. *phaseoli* or *Xanthomonas citri* pv. *fuscans.* The two pathogens are strictly related to each other, and genome transfer from Xcf to Xpp has been demonstrated. The host range of Xpp/Xcf includes other leguminous species, such as *Calopogonium* sp., *L. purpureus, Macroptilium lathyroides, Phaseolus acutifolius*, *P. coccineus, P. lunatus, Pisum sativum*, *Pueraria sp.,* and *Strophostyles helvola*, as well as several *Vigna* species, including *V*. *aconitifolia*, *V*. *angularis*, *V*. *mungo*, *V*. *radiata*, *V*. *umbellata*, and *V*. *unguiculata* [122,123]. The definition of *V. unguiculata* as a host of Xpp/Xcf is debated in the literature; indeed, a specific *Xanthomonas*, named *X. c*. pv. *vignicola*, is reported to affect *V. unguiculata*. The symptoms of CBB are visible on all aerial parts of the bean plant: leaves, pods, seeds, and stems (Figure 10). In fact, on the leaves, it is possible to observe the typical progression of the bacteria, which enter the internal leaf tissues through the hydathodes (Figure 11). Parenchymatic infection on the leaves appears as brown spots surrounded by chlorotic halos, which are difficult to distinguish from the symptoms of *Pseudomonas savastanoi* pv. *phaseolicola*. The presence of exudates can be visible on the lower sides of the leaves, and reddish streaks on the stem are peculiar to this disease. In severe infections, pods and seeds can also show brown spots. Regarding the disease cycle, the main source of the inoculum is represented by seeds that are contaminated on both the internal and external sides of the coat or inside the embryo [124]. The pathogen can survive in the seeds for about 30 years, and only 1 seed in a lot of 10,000–30,000 seeds is necessary to cause an outbreak in the field. It has been observed that 100 bacterial cells can be sufficient to transmit the pathogen from the seed to young plants. The storage of Xpp/Xcf in plant debris has been ascertained, but the time of survival can vary depending on environmental factors. The pathogen can be spread across short distances due to contaminated water movement (droplets, squash, rainfall, and irrigation) from infected debris or plants, or over long distances during the transport of infected seeds for global trade. The colonization of tissues starts in an epiphytic phase, in which the bacteria survive on the leaf surface without infecting the host until the occurrence of the appropriate humidity and temperature conditions. In this phase, the aggregation of bacterial cells in biofilms is crucial for the success of the process. Then, the bacteria enter through the hydathodes, stomata, or mechanical damage in the plant epidermis and reach parenchymatic and vascular tissues. Seed contamination from the infected plant is accomplished through different pathways: (i) through direct contact with plant-infected tissues (for example, during post-harvest treatments) via vascular tissues; (ii) through funicle tissues to the hilar region; and, finally, (iii) through flower organs [124].

Xpp/Xcf are mainly located at the coat level, but the embryo can also be affected. When germination starts, the bacteria colonize the parenchymatic tissues of the cotyledon, epicotyl, and hypocotyl, thus determining the infection of new plants. Seeds with a lower load of bacteria are mainly responsible for transmission; indeed, severe seed infection can lead to the rotting of the seeds (Figure 11), limiting the success of transmission [124].

### 3.2. Pseudomonas syringae pvs.

*P. syringae* is a species complex including 60 pathovars, depending on pathogenic interactions; 9 genomospecies, defined by DNA–DNA hybridization; and 13 phylogroups (PGs), based on multi-locus sequencing [126]. Seven of the PGs are closely related and possess the conventional type III secretion system (T3SS), while the remaining six exhibit greater genetic distance and possess different secretion systems [127]. T3SS determines the secretion of virulence proteins (effectors) that are injected into the hosts and are involved in the host–pathogen interaction and specificity [128]. The *P. syringae* species is composed of Gram-negative, arginine dihydrolase- and oxidase-negative bacteria that produce siderophores, levan (in the presence of sucrose), and, in many strains, syringomycin [129]. Taxonomically, it is classified as Bacteria; Proteobacteria; Gammaproteobacteria; Pseudomonadales; *Pseudomonadaceae*; *Pseudomonas*; *Pseudomonas syringae.*

The *Pseudomonas syringae* complex overwinters on plant debris and in weeds [130]; in the spring, it primarily reaches leaves and blooms through water movement (rain and squash), where the epiphyte cycle begins [131]. In the phyllosphere, it survives in an environment deficient in nutrients and is exposed to adverse external agents (UV radiation, wind, and competition with other epiphytes). To survive in harsh conditions, *P. syringae* recovers in advantageous niches, such as stomata and trichomes, or closes the veins and produces surfactants or biofilms that are able to protect its own cells. When humidity increases and the temperature reaches the optimal value, the bacteria multiply aggressively and enter the plant tissue, causing disease. The pathogenic cycle of *P. syringae* develops in the apoplast, which the bacteria reach after penetrating the stomata or mechanical wounds caused by insects, wind, freezing, or human activities. It has been classified as a hemi-biotrophic pathogen because, when exposed to high humidity and optimal temperature, it affects the plants and aggressively multiplies, leading to cell death and necrotizing host tissues [132].

**Figure 12:**
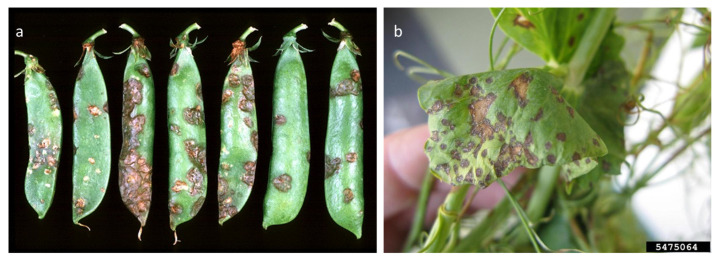
Symptoms of *Pseudomonas syringae* pv. *pisi* on pea plants (**a**) pods and (**b**) leaves. Photos released by Plant protection service and Mary Burrows, Montana State University, bugwood.org, published in PlantwisePlus Knowledge Bank, CABI Digital library [135].

*P. syringae* pv. *pisi* (Pspi) and *P. syringae* pv. *syringae* (Psy) are the causal agents of bacterial blight and of bacterial brown spot, respectively. Pspi has been recorded in 70 countries and on five continents. It belongs to PG2 of *P. syringae* [126] and, together with Psy, has a life cycle typical of this species, which includes an epiphytic phase and pathogenic behavior in plant tissues. The bacteria are seedborne and seed-transmitted, and the seed is the main source of the inoculum. They can be stored in plant debris and weeds (Psy) and be spread with water movement [133]. The two *Pseudomonas* strains thrive in cold environments, and, when the humidity increases, they penetrate tissues through lesions, mainly those caused by frost. The Pspi pathovar is classified into seven races based on the response to a differential set: races 2 and 6 are globally distributed, and race 3 is predominant in Australia [133], while races 2, 3, 4, and 5 and a new pattern were found in Spain. In addition to bacterial blight, pea can be affected by the bacterial brown spot caused by Psy. The two bacteria produce a similar symptomatology in pea: water-soaked spots appear on the leaves and stem and thereafter become necrotic (Figure 12). Deep Pspi lesions on the stem can cause the death of the host [134].

### 3.3. Pseudomonas savastanoi pvs.

The taxonomy of *P. syrinage* and *P. savastanoi* is continuously debated. Taxonomically, in the EPPO database, they are classified as Bacteria; Proteobacteria; Gammaproteobacteria; Pseudomonadales; *Pseudomonadaceae*; *Pseudomonas*; *Pseudomonas savastanoi.* Marques et al. (2000) [136] studied the genetic relationships among genomospecies 1 and 2 of *Pseudomonas* species. The results obtained through BOX-PCR and DNA–DNA hybridization suggested that genomospecies 2, including *Pseudomonas savastanoi* pv. *glycinea* (Psg), *P*. s. pv. *phaseolicola* (Psp), and *P. savastanoi* pv. *tabaci*, should be separated at the species level by genomospecies 1, which also includes Psy. Subsequently, Almedia et al., after multi-locus and genome sequencing, allocated Psy to PG2 and Psp to PG3 [127]. They demonstrated a certain diversity of bean isolates from non-bean isolates in either PG2 or PG3, but they also assessed that there are genetic similarities among bean-pathogenic strains of PG2 and PG3, suggesting connections between the two of them [127]. *P. siringae* pv. *phaseolicola* is frequently used as a synonym of *P. savastanoi* pv. *phaseolicola*, but the latter name continues to be preferred by the main international organizations dealing with phytosanitary measures and seed health (European Plant Protection Organization, International Seed Testing Association, and International Seed Federation).

**Figure 13:**
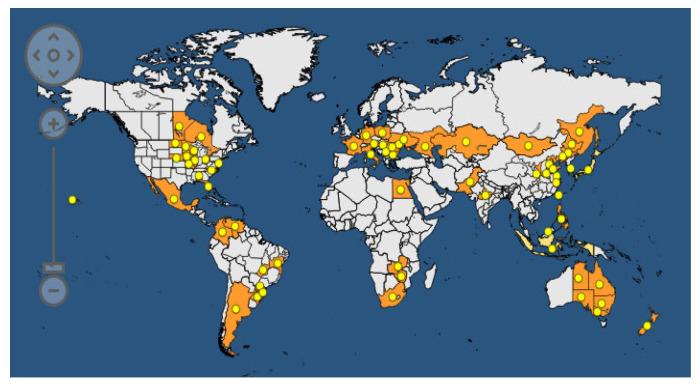
Global distribution of *P. savastanoi* pv. *glycinea.* The countries affected by Psg infections are reported in orange; the yellow dots represent the permanent presence of the pathogen in the country. EPPO Global Database.

Psg is the causal agent of bacterial blight of soybean (BBS), and it is reported in 73 countries across five continents (Figure 13) [121]. The pathovar is divided into nine races depending on how they react to different cultivars, with race 4 being the most common worldwide. *Glycine max* L. represents its specific host, and Psg is considered the most dangerous pest for this crop, leading to losses of ca. 40% at harvest. The symptoms of BBS are expressed on all upper parts of plants, affecting leaves and pods as typical oily spots surrounded by chlorotic halos, which subsequently coalesce by forming a merged necrotic spot [137]. The seed quality, oil content, and germination can be seriously compromised. The main vehicle of the pathogen is the seed, which can be contaminated either externally or internally, but weeds and plant residues can also be Psg vectors.

**Figure 14:**
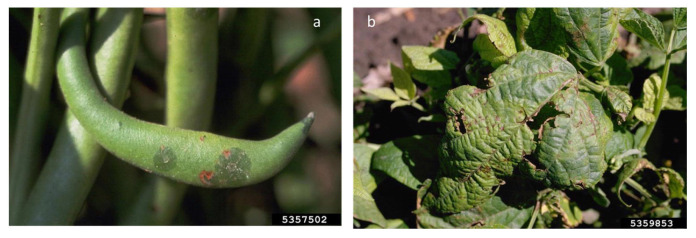
Symptoms of *Pseudomonas savastanoi* pv. *phaseolicola* on (**a**) pods and (**b**) leaves of common bean plants. Photos licensed under a Creative Commons Attribution 3.0 License, supplied by Howard F. Schwartz, Colorado State University, Bugwood.org [95].

Psp is one of the most studied bacterial pathogens, and it was the first plant pathogenic bacterium in which the T3SS was described. It is responsible for the halo blight of beans, and it is spread wherever the host is cultivated (more than 50 countries on five continents) [138]. The Psp pathovar is divided into nine races identified on the basis of the response on a differential set as a consequence of the gene-to-gene response; among races, race 6 is the most frequently detected. Halo blight symptoms appear on all aerial parts of the plant as water-soaked lesions on leaves and cotyledons with light-green halos; leaf curling and deformation; leaf, stem, and pod necrosis; and stem cracking with the emission of bacterial exudates (Figure 14). Vascular infections of seeds occur through the vascular system, and Psp can localize in the embryo, under the tegument, or externally on the seed surface. Symptoms on seeds can be visible as wrinkling and buttery spots [139], and the nature of this seedborne and seed-transmitted pest contributes to its dissemination over long distances. In humid and cold environmental conditions, Psp produces damages of up to 80–100% in cultivation [140], and, for this reason, only healthy seeds can be used as propagative materials in the US; moreover, the bacterium is included in quarantine organisms in Israel and, since 2021, also in China [121]. The typical chlorosis (halos around necrotic spots) is due to the secretion of the non-host-specific phaseolotoxin, stimulated at 18–20 °C, which inhibits the enzyme responsible for the conversion of ornithine to citrulline in the arginine biosynthetic pathway. Above 28 °C, the toxin is not detected, but non-toxigenic pathogenic strains were found to be responsible for the disease in the field [141]. Other hosts described for this bacterium are *Phaseolus acutifolius*, *P. coccineus*, *P. lunatus*, *Cajanus cajan*, *Centrosema* sp., *Desmodium* spp., *Glycine maxima*, *Lablab purpureus*, *Lens culinaris*, *Macroptilium atropurpureum*, *Neonotonia wightii*, *Pachyrhizus erosus*, *Pisum sativum*, *Pueraria lobata*, *P. thunbergiana*, *Vigna angularis*, *V. radiata*, and *V. unguiculata* [142].

### 3.4. Pseudomonas viridiflava

*Pseudomonas viridiflava* (Pv) is a Gram-negative, fluorescent bacterium. Taxonomically, it is classified as Bacteria; Proteobacteria; Gammaproteobacteria; Pseudomonadales; *Pseudomonadaceae*; *Pseudomonas*; *Pseudomonas viridiflava.* It exhibits endophytic, epiphytic, saprophytic, and phytopathogenic behavior and is phylogenetically included in the *P. syringae* complex in genomospecies 6, and it is diffused in PG7 and PG8. Its ability to cause soft rot on potato tubers, its phenotypic phase variation, and its noncanonical T3SS are typical features of PG7, while strains belonging to PG8 produce a typical toxin when tested on *Geotricum candidum*. Since 2000, Pv has been found to be responsible for 13 outbreaks and has affected about 50 hosts, classifying itself as a generalist plant pathogen. In legumes, the source of the inoculum can be represented by contaminated environments, such as biofilms, irrigation water, and rain, and can be transferred to plants by droplets, aerosols, and wind. The bacteria enter the plant tissues through stomata, hydathodes, and mechanical wounds. The infection process is favored in stressed plants by high humidity, intensive rainfall, and low temperatures [143]. Pv (PG2) was reported in 2019 on alfalfa as responsible for stem blight [144], root rot, and yellowing [145].

### 3.5. Curtobacterium flaccumfacies pv. flaccumfaciens

**Figure 15:**
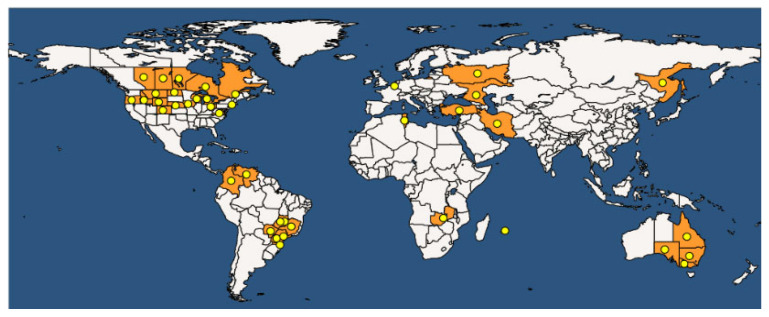
Global distribution of *Curtobacterium flaccumfaciens* pv. *flaccumfaciens*. The countries affected by Cff infections are reported in orange; the yellow dots represent the permanent presence of the pathogen in the country. EPPO Global Database.

*Curtobacterium flaccumfacies* pv. *flaccumfaciens* (Cff) is a Gram-positive bacterium that is responsible for bacterial wilt or tan spot in common bean, cowpea, mung bean, and soybean. The EPPO database taxonomically reports Cff as Bacteria; Actinobacteria; Micrococcales; *Microbacteriaceae*; *Curtobacterium*; *Curtobacterium flaccumfaciens* pv. *flaccumfaciens*. According to the EPPO Global Database, Cff was recorded in 43 countries on five continents (Figure 15), with a massive presence in the Americas (mainly Brazil and Canada), where the highest economic impact is registered.

It was detected sporadically, and subsequently eradicated, in Europe (Spain, Serbia, Switzerland, Germany, and Russia), but other non-official reports are documented in European countries. In Asia, the only affected country is Iran. In general, the most important outbreaks have occurred since the beginning of the 21st century.

**Figure 16:**
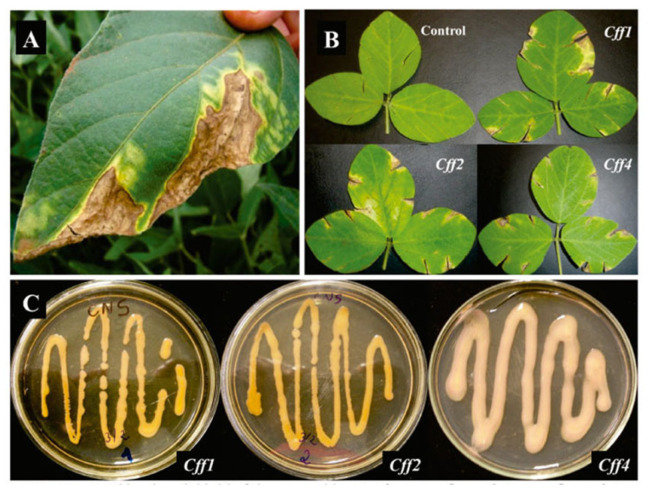
Cff (**A**) symptoms on leaves, (**B**) symptoms obtained in lab tests and (**C**) colony morphology on CNS medium. Photos published by Moreira Soares et al., 2013 [146].

The main symptoms of the disease on common bean are cotyledon and leaf wilting, which progress during the colonization of plant tissues. As generally occurs in vascular diseases, in the initial phase of the infection, the basal leaves wilt, but, after watering, they recover turgidity. As the disease progresses, watering does not allow the leaves to recover normal turgidity, and the leaves turn yellow and irreversibly wilt. With the worsening of the disease, the leaves necrotize, with lesions surrounded by chlorotic halos (more evident in cowpea), and the whole plant wilts until it dies off. The seeds can also be symptomatic, showing external yellow, orange, or red discoloration due to the presence of bacterial biofilms; on the contrary, symptoms are not visible on the external surfaces of pods (Figure 16). Germination from infected seeds is limited, and the generated plants die at a high incidence: the seedling does not always show severe symptoms in the first days post-emergence. These symptoms can vary based on the hosts: for instance, from a long-distance view, infected mung bean and cowpea fields show yellow areas that are brownish in soybean and common bean; moreover, in mung bean and soybean, a typical tan discoloration and light wilting are observed on leaves (Figure 16). Finally, in common bean and cowpea, defoliation can occur. The epidemiology of this disease is similar to that related to *Xanthomonas* or *Pseudomonas* infections. The bacteria can overwinter in plant debris, soil, or weeds or can survive for long periods (for 24 years) in seeds, under the seed coat; it is transmitted from residues or infected plants through water (rain, irrigation, and squash), reaching wounds on plant tissues either in the aerial parts or in the roots (root-knot nematodes can favor the entry of the pathogen). Additionally, Cff can enter the ovules through the funiculus or the micropyle and reach the seed, but the contamination of the pods is hampered by the vascular system closing during ripening. Seed infection is considered the main pathway for transmission to plants, with an efficacy that has been evaluated to range from 6 to 70%. Colonies of Cff isolated in 1950–1960 from diseased plants have a yellow or orange color, and only in 2007–2008 were pink and red colonies observed. Even though yellow and orange isolates appear to be more virulent on bean and cowpea than red and pink ones, all the isolates were thought to belong to the same species and pathovar following biochemical, physiological, and molecular characterization. Molecular tools can distinguish among *C. flaccumfaciens* pathovars. Combining the results of AFLP, rep-PCR, and pulsed-field gel electrophoresis (PFGE), it is possible to divide the differently colored isolates into two clades: yellow, orange, and pink strains fall into clade A, while, in clade B, only yellow strains are allocated. With the same techniques, it is possible to distinguish among pathovars: AFLP and rep-PCR recognize almost all the pathovars, but the use of PFGE, followed by digestion with enzymes, perfects the pathovar identification.

## 4. Seedborne Viral Pathogens of Principal Leguminous Crops

### 4.1. Overview

Viruses are obligate, acellular agents that reproduce inside living cells, spread between tissues, and frequently cause diseases in plants. Among all plant viruses, about one-quarter are transmitted through seeds [147]. Since viruses can survive for a long time in seeds, it is possible for a seedborne virus to spread commercially over great distances [147]. Two main pathways of seed transmission can be distinguished:

- Seed contamination: The virus remains on the coat of the seed, and infection occurs mainly through wounds of plantlets during emergence. Viruses harbored externally can be readily inactivated by a number of treatments to totally or partially eliminate seedborne infections. This type of seed transmission is specifically associated with tobamoviruses (tobacco mosaic virus—TMV; tomato mosaic virus—ToMV; tomato brown rugose fruit virus—ToBRFV) and also with some potexviruses, such as Pepino mosaic virus (PepMV) [148,149], usually with a low transmission rate.
- True seed transmission: The virus resides within the tissues of the embryo. The developing embryo can be infected either prior to fertilization (by gametic transmission) or after fertilization (by direct invasion). The latter type of transmission is more common.

The percentage of contaminated seed and seed transmission rates can be impacted by a variety of parameters: (i) virus epidemiology and genomic features; (ii) host–pathogen interaction; (iii) the location of the seed in the plant; (iv) the age of the seed; (v) environmental conditions; (vi) host resistance [150,151]. Viral diseases are major biotic constraints to legume production, especially in tropical and subtropical areas [152,153]. Cultivated food legumes are susceptible to natural viral infections due to 168 species belonging to 39 genera and 16 families [154,155]. The genomes of viruses infecting legumes can be single-stranded RNA or single-stranded DNA. An estimated 50% of legume viruses are seedborne; most of them are localized in the cotyledon/embryo, resulting in a true seed transmission pathway [156] that can result in a rate of transmission of up to 100%. Seed transmission is considered an effective method of virus introduction into a crop, as well as an excellent means of survival and dispersal, which is further boosted by insect vectors. Subsequently, the onset of vector activity and population growth in the field affect the secondary virus spread and crop losses [157,158,159]. Among all species belonging to the *Fabaceae* family, in this review, attention is focused on seed-transmitted viruses affecting both annual cool- and warm-season legumes: chickpea (*Cicer arietinum* L.), naturally infected by 36 pathogenic viruses belonging to 24 genera of 11 families [154,160,161] (at least 4 seed-transmitted species); faba or faba bean (*Vicia faba* L.), including 40 species, 24 genera, and 11 families [154,160,162,163,164,165,166,167] (at least 6 seed-transmitted species); lentil (*Lens culinaris* Medik.), including 24 species, 16 genera, and 9 families [154,160] (at least 7 seed-transmitted species); field pea (*Pisum sativum* L.), including 42 species, 18 genera, and 11 families [168,169,170,171,172,173] (at least 8 seed-transmitted species); common bean (*Phaseolus vulgaris* L.), including 83 species, 24 genera, and 12 families [154,174] (at least 9 seed-transmitted species); soybean (*Glycine max*), including 35 species, 16 genera, and 12 families [154,175] (at least 5 seed-transmitted species); cowpea (*Vigna unguiculata* (L.) Walp.), including 40 species, 16 genera, and 11 families [154,176] (at least 7 seed-transmitted species); and groundnut or peanut (*Arachis hypogaea* L.), including 27 species, 11 genera, and 10 families [154,177] (at least 6 seed-transmitted species). In addition, this review includes forage legumes: alfalfa (*Medicago sativa* L.), including 36 species, 24 genera, and 11 families (Samac et al., 2016) (at least 4 seed-transmitted species), and clovers (*Trifolium* spp.), including 32 species, 15 genera, and 10 families [178,179] (at least 4 seed-transmitted species) (Table 2).

### 4.2. Alfalfa Mosaic Virus

Alfalfa mosaic virus (AMV) (taxonomic position: family: *Bromoviridae*; genus: *Alfamovirus*; species: *Alfalfa mosaic virus*) was described in alfalfa (*Medicago sativa* L.) in 1931 and later in pea (*Pisum sativum* L.) in 1933 [180,181]. The virus has natural and experimental hosts that belong to nearly 700 plant species in 70 families [182,183]. AMV is currently found worldwide and has a wide distribution (EPPO). Alfalfa is probably the main source of inocula, but the virus is reported to also infect natural hosts including bean, faba bean, soybean, red and white clovers, chickpea, and lentil [184]. Generally, AMV is considered a major pathogen only in alfalfa [185,186], but, in Australia and in the USA, it was sporadically detected on chickpea, lentil, lupin, and soybean [180]. AMV consists of a tripartite genome containing three single-stranded RNA species and a fourth sub-genomic RNA from which the coat protein is translated. The size and shape of virions can range from spherical to bacilliform, with a diameter of 18 nm and a maximum length of 56 nm. Transmission requires the presence of RNA-1, consisting of 3644 nucleotides (nt), RNA-2 (2593 nt), and RNA-3 (2037 nt), together with the coat protein or RNA-4 (881 nt). Serologically, AMV is unrelated to any other known virus species. The virus is transmitted in a non-persistent manner by at least 14 different aphid species but can be introduced in fields from infected weeds. The effectiveness of the transmission of AMV through seeds is up to 50% in alfalfa, 5% in lentil, 10% in chickpea, and, 5% in bean [187]. The symptoms induced by AMV are generally in the form of systemic mosaic and mottle, with plant size reduction and stunting; in lentil and chickpea, it can induce necrosis along the leaflet margins and apex. Flowering and pod formation are reduced, and seeds are small and shriveled.

### 4.3. Bean Yellow Mosaic Virus

**Figure 17:**
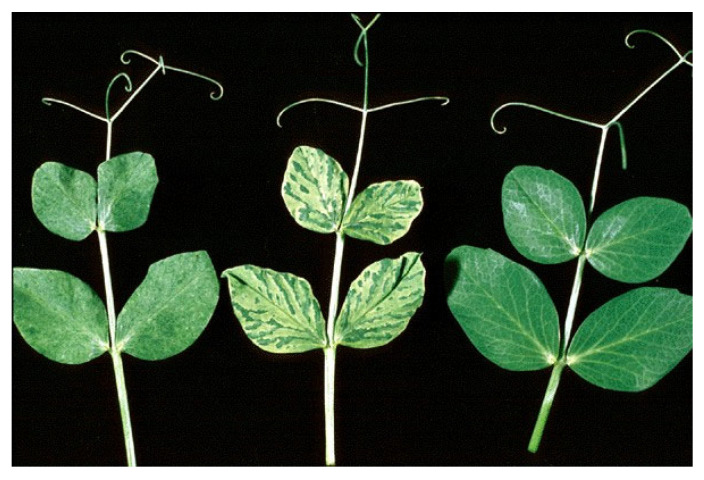
Symptoms of BYMV on pea plants. Photos released by L. Bos published in PlantwisePlus Knowledge Bank [193].

Bean yellow mosaic virus (BYMV) (taxonomic position: family: *Potyviridae*; genus: *Potyvirus*; species: *Bean yellow mosaic virus*) was discovered in common bean (*Phaseolus vulgaris* L.) in 1925, and it was the first pathogenic virus reported in chickpea (1956) [188,189]. BYMV is distributed worldwide, including in several Mediterranean countries. Many different isolates of the virus occur in economically important leguminous crops (lentil, common bean, pea, and faba bean), but minor species can also be affected [190]. BYMV is a flexuous rod-shaped virion 750 nm in length containing single-stranded RNA, with a coat protein of 282 amino acids. The genome is monopartite with 10,000 nucleotides that code for a polyprotein [191]. BYMV is serologically related to clover yellow vein virus (ClYVV), which also infects many legume species (the BYMV coat protein shares 68–76% amino acidic identity with ClYVV). On the other hand, BYMV shares less similarity with legume-infecting potyviruses such as bean common mosaic virus, bean common mosaic necrosis virus, pea seedborne mosaic virus, and soybean mosaic virus. BYMV has a broad host range of over 200 plant species, predominantly legumes. More than 20 different aphid species spread the virus in a non-persistent way from infected perennial and weed plants. Clover (*Trifolium* spp. and *Melilotus albus*), vetch (*Lathyrus* spp.), and alfalfa (*Medicago sativa*) are common field sources of the virus, while, among cultivated legumes, faba bean and pea represent the favored hosts of aphids that act as foci for the virus. BYMV is also seed-transmitted in most temperate pulses, including bean (7%), faba bean (up to 9%), field pea (10–30%), lentil, and lupin [192]. Consistent with other potyviruses, BYMV is also mechanically transmissible. Symptoms produced in response to BYMV infection may vary depending on the time of infection, the plant variety, and the virus strain. Generally, symptomatology includes mosaic, mottling green vein banding, and chlorosis (Figure 17); twisting and curling of the leaves can occur. Flowering and pod formation are reduced, and consequently, few seeds are produced, especially in cases where the incidence of BYMV is high.

In severe infections in faba bean, stem and tip necrosis and early death may occur. Crinkling, downward cupping, yellow mottling, and dead areas along the veins of infected leaves can be observed in bean. Measures to control BYMV include the management of the sowing date, spraying with mineral oils, soil mulching with reflective polyethylene sheets, and crop isolation from known overwintering disease sources [184].

### 4.4. Cucumber Mosaic Virus

**Figure 18:**
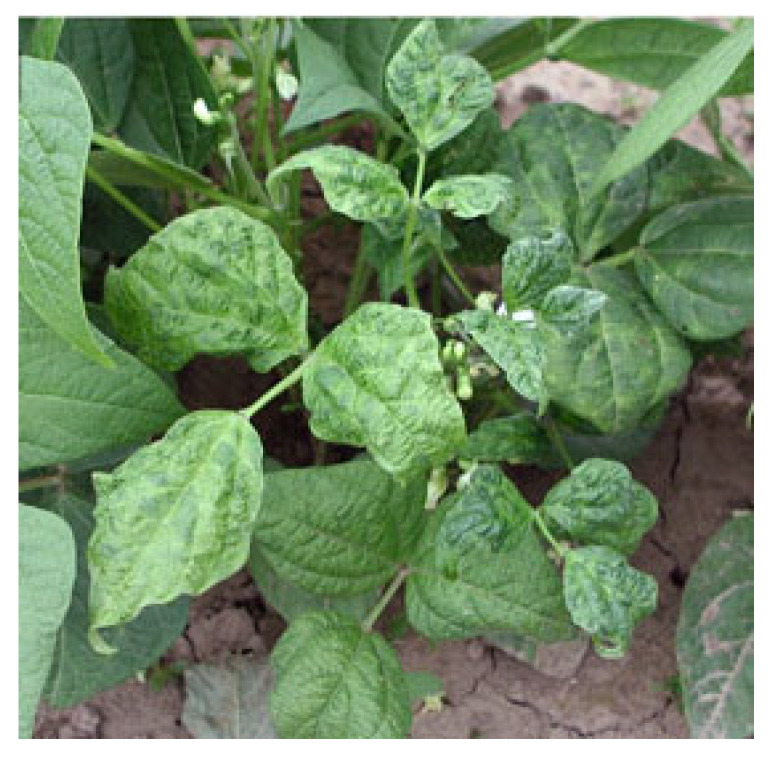
Symptoms (mosaic) of CMV on common bean plants. Photo published by Zitter and Murphy, 2009 [198].

Cucumber mosaic virus (CMV) (taxonomic position: family: *Bromoviridae*; genus: *Cucumovirus*; species: *Cucumber mosaic virus*) was first described in 1916 (Doolittle and Jagger), and it is distributed worldwide in both temperate and tropical climates, affecting agricultural and horticultural crops of 1000 plant species in 85 families [180]. Despite it representing one of the major viruses in cucurbits, in recent years, it has increasingly been reported as the causal agent of epidemics in major leguminous crops in the tropics [194] and southern Europe [195]. CMV has a tripartite genome encapsidated in isometric particles 29 nm in diameter that contain RNA-1 (3389 nt), RNA-2 (3035 nt), or RNA-3 (2197 nt). The sub-genomic RNA-4 is also encapsidated in the particles that contain RNA-3. The coat protein consists of 218 amino acids with a molecular weight of 24.2 kDa. The species has been divided into distinct serogroups called subgroups I and II, with the latter comprising 70% of the isolates. Phylogenetic analyses have shown that subgroup I can be divided into subgroups IA and IB, both of which are distantly related to isolates belonging to subgroup II [196]. CMV is distantly related to the *Cucumovirus* species *Peanut stunt virus* and *Tomato aspermy virus*. In many weed species, as well as in green vegetation and crops in temperate zones, the virus can survive from year to year. Even if they show no symptoms, perennial legumes such as clover and alfalfa can serve as easy sources of inocula. Over 80 distinct aphid species can spread the virus non-persistently [194,195]; *M. persicae* and *A. gossypii* are two of the most prevalent aphid vectors among them. Mechanical inoculation and transmission through seeds are further known routes of virus spread. The rate of seed transmission is reported to be from 10% to 100% in bean, from 50 to 90% in soybean [192], and from 7 to 64% in lentil, whereas in other hosts, such as chickpea, it is erratic and can vary from 0.1% to 1%, occasionally reaching 2% [181]. The CMV symptomatology pattern varies with the host [194]. In lentil, symptoms include plant chlorosis, leaf malformation, and stunting; in chickpea, internode reduction, shoot proliferation, and bushy stunting occur (Figure 18). Finally, mild mosaic and severe plant malformation were observed in bean. Pods can be reduced in number or only partially filled with seeds, resulting in yield losses, whose incidence ranges from 5% to 75% depending on the cultivar, age of infection, virus strain, and environmental conditions [197].

### 4.5. Pea Seedborne Mosaic Virus

Pea seedborne mosaic virus (PSbMV) (taxonomic position: family: *Potyviridae*; genus: *Potyvirus*; species: *Pea seedborne mosaic virus*) was first identified in Europe in 1966 and then in Japan in 1967. It was subsequently discovered in the US in 1969 and in New Zealand in 1980 [180]. PSbMV is now found throughout the world. The virus naturally infects lentil, pea, faba bean, and other legumes, and it is a minor issue in chickpea [180]. Because of the potential for high rates of seed transmission and the international trade of breeding lines, PSbMV continues to be a very serious disease in many pulse crops, particularly under predisposing environmental conditions. This virus consists of flexuous rod-shaped particles with a modal length of ca. 770 nm. The genome consists of monopartite single-stranded positive-sense RNA of about 9.9 kb, coding for a polyprotein of approximately 334 kDa in size that is cleaved into various functional proteins by viral proteases. PSbMV has a distant serological relationship with *bean yellow mosaic virus*. Pathotypes designated P-1, P-2 (also called lentil strains), P-3, and P-4 have been distinguished by the susceptibilities of different lines of pea and based on sequence variability in two coding regions of the RNA [199]. More than 20 aphid species can transmit PSbMV in a non-persistent manner; however, the spreading of the pathogen in new areas can also be associated with the global trade of breeding lines and with its seedborne nature. The establishment in these areas is further favored by vectors, if present. Moreover, the ongoing usage of contaminated seeds, which are collected at the end of a season and then utilized in successive plantings, is a significant contributor to PSbMV’s persistence. Pea (up to 90%) and lentil (up to 44%) have been shown to have high seed transmission rates; however, other hosts such as chickpea (1%) and faba bean (3%) may show lower transmission efficacy. A wide range of symptoms are associated with PSbMV infection. They consist of mosaic, vein clearing, leaf rolling, chlorosis, necrosis of shoot tips, and plant stunting. Pods may be deformed and fail to set, and seed production can drastically decrease (by up to 77% in lentil). Virus-infected seeds may be abnormal with low germination. Symptoms may vary due to cultivar differences, different virus strains, the occurrence of mixed infections (with PEMV), and environmental effects. Symptoms are typically more severe in the case of seedborne infections [184].

### 4.6. Other Impacting Viruses Limited to Specific Hosts

The following part discusses two cases of viruses that, based on their epidemiological characteristics and effects on yield losses, are regarded as severe threats despite only having a few hosts (bean and soybean).

#### 4.6.1. Bean Common Mosaic Virus and Bean Common Mosaic Necrosis Virus

**Figure 19:**
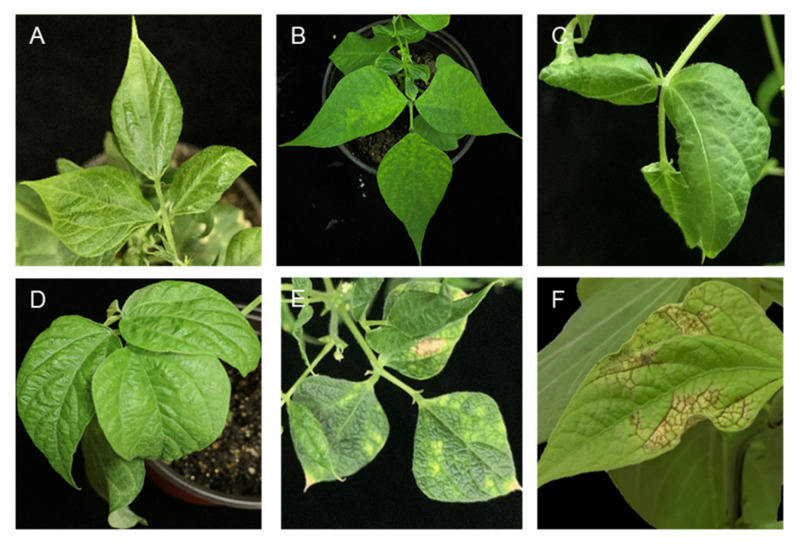
(**A**–**F**) Symptoms of BCMV on common bean plants. Photos published in Tang and Feng 2023 [205].

Bean common mosaic virus (taxonomic position: family: *Potyviridae*; genus: *Potyvirus*; species: *Bean common mosaic virus*) and bean common mosaic necrosis virus (taxonomic position: family: *Potyviridae*; genus: *Potyvirus*; species: *bean common mosaic necrosis virus)* are two potyviruses considered to be the most important viruses causing diseases in common bean [200]. While BCMNV is restricted to Africa, Europe, North America, and South America, BCMV is widespread and is documented in all locations where legumes are grown (EPPO). BCMNV and BCMV are formally classified as separate potyvirus species (ICTV), despite the fact that BCMNV was once believed to be a serotype of BCMV. It is thought that in Central or Eastern Africa, BCMNV evolved from BCMV [201]. BCMV and BCMNV have a negative impact on both the commercial-scale cultivation of this high-value crop and production by smallholder farmers in developing nations where beans represent a significant source of dietary protein and minerals. Yield losses due to BCMV and BCMNV may vary between 6 and 98%, depending on the cultivar, the time of infection [201], and the virus strain [202,203]. Both viruses have flexuous, filamentous particles ca. 750 nm long and 12–15 nm wide and a genome monopartite structure with 10,000 nucleotides that code for a polyprotein [191]. Both BCMV and BCMNV are mechanically transmitted, and several aphid species (e.g., *A. pisum*, *A. fabae*, and *M. persicae)* act as vectors in a non-persistent manner. The rate of seed transmission for both viruses has been shown to be 35% [191], and it significantly contributes to the first crop infection. In susceptible bean genotypes, BCMV and BCMNV induce similar symptoms, including mosaic, dwarfing, chlorosis, and leaf curling (Figure 19) [204]. The intensity and severity of the symptoms depend on various parameters, including the strain, the bean cultivar, and the plant age. The difference between the two viruses relies on the phenotypes generated in resistant cultivars [200].

#### 4.6.2. Soybean Mosaic Virus

Soybean mosaic virus (SMV) (taxonomic position: family: *Potyviridae*; genus: *Potyvirus*; species: *Soybean mosaic virus*) [175] was first observed in the US and documented in the Western scientific literature by Clinton. In soybean, more than 100 viruses are reported, and among them, SMV is considered a major threat [206] since it is able to cause serious yield losses and the deterioration of seed quality. SMV is present in all soybean-growing areas of the world [207]. In contrast to its wide distribution, this virus has a very narrow host range; indeed, it is reported to infect cultivated soybean (*Glycine max*), its wild relative (*G. soja*), and a limited number of natural hosts [208]. The virus particle is a long and flexuous rod. The genome consists of single-stranded, positive-sense, polyadenylated RNA of approximately 9.6 kb with a virus-encoded protein (VPg) linked at the 5′ terminus. Yield losses due to SMV range between 8 and 25%, depending on the infection time, and only when the plant is co-infected with bean pod mottle virus can the percentage increase up to 66–86% [175,209]. SMV shows significant variability between strains based on symptomatology and on the cultivars that they can infect [175]. SMV is transmitted by over 30 species of aphids in a non-persistent manner, including *Aphis glycines*, whose flight is correlated with the incidence of the disease [210]. The infection rate of SMV in soybean seeds is higher if the plant is infected before flowering [211,212]. Plants originating from infected seeds can serve as the initial points of inocula in a field with secondary infections resulting from aphid feeding. Seed infection can be as high as 75% depending on the soybean cultivar and the strain of the virus, but it is usually less than 5% (Table 3) [175]. Infection with SMV may result in smaller, less vigorous seeds with lower oil but increased protein and amino acid contents [213,214]. Foliar symptoms vary from moderate to severe leaf mottling, the distortion of leaves, necrosis, overall stunting, and, occasionally, the death of the plant. As for most plant viruses, the extent of crop damage is dependent on the host genotype, the predominant virus strain, infection incidence, environmental conditions, and the developmental stage at which soybean plants become infected [207].

## 5. New Reports in Leguminous Crops

In the last few decades, the climate emergency and the improvement in global trade have fostered the spread of novel seedborne pathogens [215,216]. A study conducted in Nicaragua on common bean, aiming to establish seed health, revealed the presence of fungi not recorded before in this plant species. Among the new pathogens, *Lasiodiplodia theobromae*, which is a non-specific pathogen with a wide host range and is responsible for fruit rot, root rot, dieback, and cankers [217], was found. In pathogenicity tests, *L. theobromae* caused dieback, decay, cankers on stems, and plant decline [218]. In the same work, *Corynespora cassiicola*, which is usually associated with the target spot of soybean and cotton, was also reported in common bean seeds. In this case, dark or brown-dark lesions on stems, softer and thinner stems, root rot, and blight were reported as experimental symptoms. The authors also found *Colletotrichum gleosporioides*, known as the causal agent of anthracnose of the olive tree, and *Colletotrichum capsici*, which is linked to anthracnose of pepper [219]. Pathogenicity tests conducted on common bean revealed that *C. gleosporioides* is able to induce necrosis, brown lesions, spots, cankers on stems, seed rot, soft stem and leaf blight, dieback, and small dark spots on the stem, while *C. capsici* is responsible for the discoloration of roots and dark spots on the cotyledon [218]. Interestingly, fungi belonging to the *Botryosphaeriaceae* family were also found on the seeds of Italian common bean landraces, namely, *Botryosphaeria dothidea* and *Diplodia mutila* [17]. The pathogens were usually found in woody plants, causing the dieback of grapevines [220,221]. In common bean, *B. dothidea* and *D. mutila* were shown to inhibit germination by up to 50%, and the seedlings that developed were stunted, mainly due to root rot. When inoculated on grown plants, they were able to produce necrotic lesions that worsened over several days [17]. Similarly, *Apiospora arundinis* was isolated from the seeds of common bean, and, when inoculated into plants, typical necrotic spots on the leaves confirmed its pathogenicity in the new host [119]. Moreover, pathogenic *Sclerotinia sclerotiorum* was isolated for the first time from the seeds of faba bean and was shown to be able to induce leaf chlorosis and severe stem rot when artificially inoculated [119]. In 2022, an infection of common bean caused by *Curvularia verruculosa* was reported in China, and the symptoms were described as leaf spots [222]. The development of the novel high-throughput sequencing (HTS) platform and its routine application in plant virology can facilitate the detection of known viruses/strains along with the identification and reporting of new viruses. Because of its unbiased and hypothesis-free testing of plant samples, substantial virology knowledge is required to suitably interpret the results.

## 6. Main Methods for Seedborne Pathogen Detection

Seed health is a key factor in preventing the spread of seedborne pathogens in new environments, shedding light on the crucial role of early diagnosis. This field is tricky since most of the seeds affected by pathogens are symptomless, and concomitantly, only a low percentage of these infections will be translated into symptomatic plants [223]. For this purpose, methodologies to detect pathogens in seed lots were developed, mainly based on traditional microbiological methods and molecular strategies. Moreover, international organizations, such as the European Plant Protection Organization (EPPO), the International Seed Testing Association (ISTA), and the International Seed Federation (ISF), have set up and validated several protocols that can be used in seed certification processes. The first approach to assessing a sample is a visual examination based on symptom observations. In some cases, different pathogens can share the same symptomatology; thus, the use of a microscope is crucial. In fact, a stereoscopic microscope can be used to observe the overwintering or reproductive structures of fungi, allowing discrimination between different pathogens [224]. The most frequently used seed analyses are based on incubation methods, which are particularly helpful for high-incidence pathogens. To allow fungal growth, seeds are usually incubated in Petri dishes, on impregnated filter paper (i.e., blotting test), or on agar media. Tests typically last 2–10 days at temperatures between 20 and 28 °C, with the chosen parameters being dependent on the fungal pathogen. In **incubation methods**, to remove external contaminants, a stage of surface sterilization is frequently included. External disinfection is the first step that must be assessed on a case-by-case basis since it allows for the eradication of external saprophytic microflora that may compromise the accuracy of the diagnosis. Surface sterilization, however, may help prevent phytopathogenic microorganisms that dwell in the seed’s outermost layers from being missed during the diagnostic process. In this situation, it is important to keep in mind that internal pathogenic bacteria in the seeds pose the greatest challenge to seed health, whereas external pathogens can be easily combated by seed coating or other seed treatments.

Fungal pathogens contained in the interior layers of seeds (below the seed coat, endosperm, or embryos) are those that may develop into colonies when surface sterilization is performed. In these techniques, non-selective media, such as Potato Dextrose Agar (PDA) and Malt Extract Agar (MEA), are used, but because they do not always allow the formation of reproductive structures, less rich media, i.e., Water Agar (WA) and Cornmeal Agar (CMA), are preferable. Moreover, to facilitate the process of seedborne pathogen isolation, several selective media were developed by adding antibiotics or chemicals, such as streptomycin or bromophenol blue. The final step of these procedures involves the microscopic observation of the colonies and the detection of species-specific structures that can allow the final identification. **Incubation** is also used to detect bacteria. In this case, the protocols are based on the soaking method: seeds are soaked in an appropriate amount of buffer and macerated for 2–18 h at predefined temperatures. During this process, the bacteria are released from the seeds into the buffer, which is then plated on semi-selective agar media. This approach also enables the isolation of bacterial pathogens from the endosperm and embryos. After 5–15 days of incubation (depending on the species), colonies develop, and then bacteria can be recognized by visual observation. This stage is critical since it is influenced by the analysts’ experience, and the use of reference strains to compare with suspected colonies appears to be particularly useful. After detecting putative pathogenic isolates, confirmation assays are used to establish the identity and to verify the pathogenicity. Pathogenicity tests are performed while trying to balance between the reproduction of the natural infection process and laboratory feasibility. For details about the most used inoculation protocols, refer to the “International rules for seed testing” (https://www.seedtest.org/en/publications/international-rules-seed-testing.html, accessed on 26 January 2023) and, for Cff, to the diagnostic protocol available on the EPPO website (https://gd.eppo.int/, accessed on 26 January 2023). Once purified, colonies with the typical morphology can be identified by using molecular methods: PCR assays were developed by Tegli et al. (2002) and Guimaraes et al. (2001) to confirm the identity of Cff [225,226]; Audy and collaborators (1994) set up a robust protocol to detect Xpp/Xcf [227], and Arnold et al. (1994) designed specific primers to identify Pspi [228].

**Serological methods**, which use polyclonal or monoclonal antibodies to detect seedborne infections, can be a viable alternative. The most commonly used technique in this field is the double-antibody sandwich-enzyme-linked immunosorbent assay (DAS-ELISA). The ELISA has the advantage of being applicable to biotrophic and necrotrophic pathogens and is largely employed in the detection of seedborne viruses. Finally, molecular methods have gained popularity in seedborne pathogen diagnosis due to their rapidity, sensitivity, specificity, and ease of interpretation. These methods need a preliminary step in which fungal DNA has to be extracted and purified [229]. Many laboratories are still dependent on standard methodologies that involve the growth of monosporic or monohyphal culture, followed by lyophilization and then extraction with a standard kit based on the use of chemicals [229,230,231]. The Polymerase Chain Reaction (PCR) is the most commonly utilized molecular technology in pathogen detection, and it is also the least expensive method employed in diagnostic procedures. The most commonly used primers for fungal identification were produced more than two decades ago and are based on the nuclear ribosomal operon [232]. In fact, primers based on the Internal Transcribed Spacer (ITS), the large subunit (nrLSU-26S or 28S), and the small subunit (nrSSU-18S) are routinely used. Among these, the ITS demonstrated the fastest evolution, implying considerable variability, making this region ideal for species identification. Furthermore, the ITS can be combined with protein-coding genes to provide a more precise species identification. The ITS sequence can also be used in phylogenetic analysis. For this purpose, the commonly used regions include the largest (RPB1) and second-largest (RPB2) subunits of RNA polymerase, translation elongation factor 1-alpha (tef1), and beta-tubulin (tub2/BenA) [229]. In addition to traditional PCR, advanced molecular techniques have been developed, including quantitative PCR, nested PCR, multiplex PCR, and LAMP (loop mediated amplification). Moreover, with the advent of Next-Generation Sequencing (NGS), protocols based on RNA-Seq-based NGS can be used for the rapid identification of fungal plant pathogens inducing novel diseases [229].

In the last few decades, rapid and specific serological (enzyme-linked immunosorbent assay, ELISA) and molecular techniques (molecular hybridization and DNA amplification) have also been developed for the detection of plant viruses. For all the viruses included in this review, it is possible to apply serological and/or molecular detection methods. In pathogenic virus diagnosis, serological approaches are usually enough for regular testing, while a molecular-based detection approach can be effective for strain differentiation when high sensitivity is required. In particular, AMV can be detected by serological methods (e.g., ELISA and TBIA) [184] using polyclonal and monoclonal antibodies or molecular methods (RT-PCR), including real-time PCR [233,234]; methods available for BYMV detection include monoclonal and polyclonal [184] antibodies produced for ELISA and primers for detection by RT-PCR and real-time PCR [235,236]; and CMV can be detected by ELISA using polyclonal and monoclonal [237] antibodies and by RT-PCR/real-time PCR [233,236,238], as is the case for PSbMV (ELISA using polyclonal antibodies and nucleic acid (PCR)-based methods). Monoclonal and polyclonal antibodies were produced for BCMV and BCMNV detection by ELISA [239,240], and primers have been developed for their detection by RT-PCR [241]; only for BCMV is a real-time PCR assay available [242]. For SMV, different serological and/or molecular assays are available [243]. These methods are reported to detect the presence of these viruses, but few validated methods are reported by ISTA, EEPO, ISF-ISHI, and the National Seed Health System (NSHS). These methods are limited to detection by ELISA of PSbMV, pea early browning virus (PEBV), SMV, and bean pod mottle virus (BePMV).

## 7. Management Strategies of Legume Crops

In recent years, global seed trade has fostered the spread of seedborne diseases. Several techniques can be implemented to mitigate their effects on crops, such as reducing long-distance spread, seed health testing, breeding, and pest management in the field.

The European and Mediterranean Plant Protection Organization (EPPO) is an international organization involved in plant protection cooperation, and among its objectives is the development of an international strategy against the introduction and spread of pests in agricultural and natural ecosystems. The EPPO Global Database provides information about pathogen distribution in worldwide territories and archives pest categorization and restriction requirements. Of course, field inspection and diagnostic analyses continuously implement the information provided by EPPO GD. The categorization established in different countries for the pathogens dealt with in this review is reported in Supplementary Table S1. Awareness about phytosanitary emergencies and risks must be supported by robust seed health testing methods. In this context, seed diagnosis also plays a critical role for seed companies, since it contributes to the quality of their output, which can have a substantial impact on plant health, development, and harvesting [244]. ISTA, ISHI, and NSHS chose standard methods based on six crucial aspects: (i) specificity, defined as the ability to distinguish the target microorganism from others found in the seeds tested or in the field analyzed and to avoid false-positive results; (ii) sensitivity, defined as the ability to detect the target pathogen even at low concentrations in seed lots; (iii) speed; (iv) simplicity, outlined as the minimization of steps in a procedure and the feasibility for non-expert operators; (v) cost-effectiveness, suitable to be part of routine processes; and (vi) reliability, indicating that the test must be robust enough to ensure repeatability on the sample, between samples, and regardless of who carries out the method [245]. In this scenario, lots in which seeds are free of pathogens or that have an infection value lower than the allowable limit may be considered healthy and appropriate for planting. On the other hand, one of the most targeted strategies for reducing the impact of seedborne diseases is the research and optimization of resistant varieties. This goal can be difficult to achieve in some circumstances due to the complexities of resistance genetics and pathogen variability. For instance, in common bean alone, 100 races of *C. lindemuthianum* have been identified based on 12 differential cultivars [246]. Studies conducted on *Ascochyta* spp. in legumes highlighted heterogeneity in resistance genetics, and the use of wild cultivars as a source of genetic diversity is a promising strategy to develop resistant materials [60]. In the past, information about the resistance of common bean varieties to *R. solani* was scarce: it was defined as quantitative in nature and it was influenced by environmental conditions. In recent studies (2019), the knowledge of resistance to *R. solani* in common bean was improved: Oladzaz and coworkers analyzed a pool of varieties derived from two different geographic areas, namely, Andean and Middle American areas, and they found that 2 varieties out of 552 showed resistance to the fungus [78].

Disease resistance is also studied to counteract bacterial diseases. The breeding of resistant soybean varieties is considered to be the most reliable approach, even if the development of plant resistance is hindered by the evolution of the pathogen virulence [128]. *Avr* genes were molecularly characterized in Psp, allowing the development of bean varieties resistant to halo blight; however, no complete resistance was found to race 6, which is the most widespread [139]. The breeding strategy was also used to control Cff: seed industries dedicated their efforts to obtaining resistant materials starting from resistance found in red kidney and dark red kidney bean cultivars to yellow, orange, and purple bacterial variants. Moreover, in the USA, the bean cultivar Emerson was obtained by crossing Great Northern 1140 and PI 165078 from Turkey, but this new breeding material showed a limited cultivation area [247]. Extensive and more in-depth studies on the resistance of bean to common blight were carried out and evidenced that more genes regulate resistance, resulting in quantitative and polygenic characters. Moderate resistance exists in the Mesoamerican gene pool, and an interesting source was identified in the NOD-like receptor family, where frequent recombination sites were found at the end of the chromosome, both in Mesoamerican and in Andean common beans. Moreover, the source of resistance to both Xpp and Xcf is represented by 27 quantitative trait loci (QTL) in 11 bean chromosomes. However, the complexity of the bacterial population and the strain variability (mainly related to their differential virulence on leaves or pods) hinder the introduction of resistance genes into common bean commercial varieties. Advances in breeding, which have been achieved thanks to marker-assisted selection and new knowledge of the transcriptome, show that resistance is associated with the up-regulation of salicylic acid and the down-regulation of photosynthesis. Finally, high-resistance genes were found in *P. coccineus, P. lunatus, and P. acutifolius*, suitable to be used in intraspecific breeding research [124]. In addition to *Xanthomonas*, pathogenic *Pseudomonas* species have plastic populations, and chemical control is not currently considered a solution.

Nowadays, a consolidated and practical approach is to set up plans to control plant pathogens based on the use of chemical fungicides, biological antagonists, or a combination of both. The choice of treatment to be used in counteracting seedborne diseases is based on the pathogen’s localization in the seeds. For instance, contact fungicides can control only surface contaminants and are useless against those microorganisms that are stored in the internal tissues. On the contrary, fungicides defined as translaminar or cytotropic are also effective against slight infections in the external layers of the seeds. However, when the infection involves the inner layers of the seeds (embryos), only systemic compounds can be effective in preventing the disease [248]. Compounds frequently used for seed treatment belong to triazole, carboxin, acylalaninate, dicarboximide, benzimidazole, and strobilurins chemical groups [249,250]. In the last several years, restrictions on the use of chemicals and the increasing rise of organic agriculture have placed the focus on the development of biological control means, such as natural extracts, beneficial microorganisms, and antagonistic fungi. Among natural extracts, the essential oils of thyme and garlic have attracted great interest, as they were shown to be able to inhibit plant pathogen growth, including *C. lindemuthianum* [251]. On the other hand, several works proved that the treatment of legume seeds with *Trichoderma* spp. or Plant-Growth-Promoting Rhizobacteria (PGPR) increases the germination rate and reduces the incidence of seedborne diseases. In fact, El-Koly et al. demonstrated that coating seeds with *Bacillus megaterium*, *B. subtilis*, *Pseudomonas fluorescens*, *Serratia marcescens*, *Trichoderma album*, *T. harzianum*, *T. lignorum*, and *T. viride* strongly reduced the pre- and post-emergence damping off caused by *R. solani* and did not affect the nodulation of rhizobia [252]. Biological control has also been tested against *M. phaseolina* by using strains of *Bacillus* spp. and *Trichoderma* spp. and both were shown to be able to reduce the incidence of charcoal rot in common bean [253,254]. Similar results have been achieved in soybean during a trial in which chemical agents and biological treatments with *T. viridae* and *B. subtilis* were tested. The results highlighted the ability of the microorganisms to counteract charcoal rot, although chemical agents were shown to be more adaptable to climatic conditions [255]. In 2018, seeds of common bean treated with *B. amyloliquefaciens* showed a reduced incidence of *R. solani* root rot, along with the increased tolerance of the seedlings to drought [256]. Regarding *Ascochyta* spp., effective alternative strategies involving the combination of *Curtobacterium rosae*, thyme oil, and high temperature were shown to significantly increase seed germination and reduce *Ascochyta* presence in pea seeds [257]. Similarly, in 2016, experiments were carried out in China by testing putative biocontrol agents against *A. pinodes*: in this study, PGPR, identified as *Pantoea agglomerans*, *B. subtilis*, and *B. amyloliquefaciens*, showed strong biocontrol activity, reducing the incidence of the disease both in the greenhouse and in the field [258]. Strains of *Bacillus* have been tested to control *S. sclerotiorum* infection in common bean. In 2018, Sabatè and coworkers demonstrated that when incubating common bean seeds with *Bacillus* strain B14, the growth of bacterial contaminants and the presence of pathogenic fungi were completely inhibited. Moreover, they proved that *Bacillus* strains are also able to mitigate *S. sclerotiorum* infection in common bean seedlings [259]. On the other hand, biological formulations effective against *Botrytis* spp., traditionally controlled by crop rotations or by using fungicides, have been developed and are already present on the market. Indeed, *Gliocladium catenulatum* strain JI446 and a *B. subtilis* strain have been used to formulate two products, Prestop and Serenade, respectively, to mitigate the effects of *B. cinerea* and *B. fabae* on faba bean (*Vicia faba* L.) [260].

Integrated control strategies are also implemented for the management of bacterial diseases. Psg bacterial blight can be successfully controlled using integrated disease management. In this context, crop rotation, the use of pathogen-free seeds, the avoidance of planting in areas prone to frequent frosts or extreme wet weather, crop hygiene, and the management of sowing time can be adopted. Donmez and Alyieva tested *Bacillus* spp. and *Pseudomonas* spp. strains against Psp, obtaining a 50% reduction in infection [261]. A similar strategy has also been used against Xcf; indeed, the bacterium *Rahnella aquatilis* was shown to be able to inhibit the growth of Xcf in vitro and to induce host defense by enhancing phenolic compound synthesis and peroxidase activity in common bean plants [262]. Moreover, Sangiogo et al. used *Bacillus* and *Pseudomonas* spp., alone or in combination, against Xcf, and the results demonstrated the effectiveness of the treatments, depending on the timing of the incubation [263].

Bacteriophages are viruses that affect several plant pathogenic bacteria. They can be used as a biological control means against bacterial diseases because they are able to selectively affect pathogenic bacteria by lysing their cells. Commercial products based on phages are already available, and several research trials were conducted to counteract BBS. In 2022, phages belonging to the genus *Ghunavirus*, subfamily *Studierviriae*, were studied against Psg, and their activity in devitalizing *P. savastanoi* pv. *phaseoli* and P. s. pv. *pisi* was also demonstrated. The authors also proved that these phages were not able to infect beneficial epiphytic *Pseudomonas fluorescens*, *Rhizobium*, and *Bradyrizobium* [137]. Strains of *B*. *alcalophilus*, *B. atrophaeus*, *B. megaterium*, *B. mycoides*, *B. subtilis*, *Lysinibacillus sphaericus*, *P. fluorescens*, and *P. putida* were recently studied with promising results in an in vivo biocontrol assay on halo blight [225]. *B. cereus*, *P. fluorescens*, and *Pantoea agglomerans* were successfully used as seed treatments to control bacterial wilt caused by Cff; moreover, it was demonstrated that *P. agglomenrans* is able to colonize seedling tissues and protect the plant for 7 days post-emergence [224].

A separate discussion is presented for viruses because the control methods are strictly dependent on the way they are transmitted. Namely, the control of vectors (insects and weeds), the employment of virus-free seeds, and the use of resistant cultivars are the main strategies in virus control. AMV is both seed- and aphid-transmitted, and thus, a range of general control measures can be applied: (i) the use of virus-free seeds, (ii) the control of virus spread from overwintering hosts by spatial separation, and (iii) aphid vector control. However, insecticides have limited use, as the virus is non-persistently transmitted by aphids. Finally, no source of resistance to the virus has been reported in bean [184]. Regarding BYMV, in regions and in crops where seedborne infection is a major concern, it is advisable to use virus-free seeds. Another control measure is the isolation of the crop from weeds belonging to the host range of BYMV. Nevertheless, the maintenance of low levels of the virus in surrounding crops and weeds can be difficult because of the wide host range of BYMV. As with other non-persistently transmitted viruses, the application of insecticides to control the spread by aphids is significantly effective where the vector has built up large populations. BYMV-resistant varieties were selected for faba bean [264,265], pea [184], and lentil (ILL7163) [266]. Preventive control measures are suggested for CMV, such as transplanting plants obtained from certified virus-free seeds. Conventional methods of virus control are difficult to apply due to the wide host range of CMV, which infects many weeds and crops that can act as virus reservoirs. Since CMV is transmitted by over 80 aphid species, the diverse behavior of various aphid vectors can greatly reduce the impact of insecticide sprays, which are more effective when the insects are a direct ‘‘pest’’ rather than a ‘‘vector’’. Finally, the development of new varieties resistant to CMV, either by conventional breeding methods or by gene technology, is gaining momentum. Similarly, because infected seeds are the primary source of PSbMV, the use of certified virus-free seeds is the most effective control method, especially in areas where the virus was not previously reported. Managing aphid vectors could offer some protection by reducing aphid-mediated secondary spread from the initial disease loci. Growing resistant cultivars offer the best strategy for reducing the impact of PSbMV. In fact, the rate of seed transmission varies with the cultivar and virus strain. Additionally, for both BCMV and BCMNV, the most effective control measure is the cultivation of resistant common bean varieties. In many parts of the world, progress has been made in combating BCMV by breeding bean varieties possessing the *I* gene, a dominant gene conferring resistance to most BCMV strains. In the absence of resistant cultivars, the use of virus-free common bean seeds minimizes the risk of outbreaks.

SMV management could include the use of virus-free seeds; as the virus is non-persistently transmitted by aphids, vector controls using insecticides have limited use. The use of resistant varieties represents a feasible aim and depends on the type of resistance the plant has and on the strain of the virus [267]. Resistance is more important in late- than in early-planted soybean, because insect vectors increase in population density during the season, with SMV infection typically worse in late-planted soybeans [212].

Altogether, these findings open interesting perspectives on the biological and eco-friendly management of legume crops.

## 8. Conclusions

In conclusion, climate change and the world’s population growth have created new concerns, such as food scarcity. As a result, legumes have the potential to increase agricultural diversity and production while also contributing to the protein requirements of the human diet. In this context, successful legume management is critical in order to enhance productivity and market value. This procedure necessitates the advancement of information about these crops, including the optimal agronomic strategies for generating profits, as well as phytopathological evidence to combat plant infections. The target microorganisms in seed health assessments are chosen based on the plant species, but also on information about the presence or absence of a specific disease in the country where the samples are produced. In recent years, in addition to species-specific seedborne diseases, novel microbes, particularly fungi, have been described on a daily basis. Indeed, the use of non-selective media in seed analysis allows for the recovery of unanticipated infections, and their identification and disease description aid in preventing their spread to new areas. On the contrary, despite the recovery of novel putatively hazardous bacteria, the selective procedures utilized in bacterial analyses may result in the selection of well-known pathogens. This review compiles the most important information on seedborne infections on legumes in order to aid in their identification. Furthermore, the report of novel infections sheds light on the critical importance of an open-minded approach to seed health and provides a broad perspective on crop management based on innovative and environmentally friendly solutions.

## Figures and Tables

**Table 1:** List of seed-transmitted fungi in widespread legume crops.

	Symptoms	Transmission	Species	Host
Anthracnose (*Colletotrichum* spp.)	Necrotic stem and pod lesions Leaf vein streaks Chlorotic leaves Wilting and desiccation	Seed (seed coat and embryos) spore dispersion through atmospheric agents	*C. lindemuthianum* *C. lupini* *C. pisi* *C. truncatum* *C. cliviicola* *C. trifolii*	*Phaseolus vulgaris* L. *Lupinus* spp. *Pisum sativum* L. *Lens culinaris* Medik. *Glycine max* L. *Vigna unguiculata* L. *Medicago sativa* L.
Ascochyta blight (*Ascochyta* spp.)	Necrotic lesions on stems Necrotic spots on the leaves Seed rot	Seed (seed coat and embryos) Conservative structures in plant debris	*P. exigua* var. *exigua* *A. rabiei* *A. pisi* *A. fabae* *A. lentis* *A. phaseolorum* *A. sojicola* *P. sojicola* *A. imperfecta*	*Phaseolus vulgaris* L. *Cicer arietinum* L. *Pisum sativum* L. *Vicia faba* L. *Lens culinaris* Medik. *Vigna unguiculata* L. *Glycine max* L. *Medicago sativa* L.
Charcoal rot (*Macrophomina phaseolina*)	Charcoal rot of crown Root rot Seed rot	Seed (seed coat) Sclerotia conserved in root and stem debris	*M. phaseolina* *M. pseudophaseolina*	*Phaseolus vulgaris* L. *Cicer arietinum* L. *Pisum sativum* L. *Vicia faba* L. *Vigna unguiculata* L. *Glycine max* L *Medicago sativa* L *Lens culinaris* L.
Root rot, crown rot, and seed rot (*Rhizoctonia solani*)	Hypocotyl, crown, stem, and root rot, blights; wire stem; and damping off Seed rot and decay, pre-emergence damping off, and missing stand	Seed (seed coat) Sclerotia conserved in soil Dispersion of basidiospores through atmospheric agents	*R. solani*	*Phaseolus vulgaris* L. *Lupinus* spp. *Pisum sativum* L. *Lens culinaris* *Glycine max* L. *Vigna unguiculata* L. *Medicago sativa* L.
*Diaporthe/Phomopsis* complex	Seed decay, seed rot, pod and stem blight, and stem cankers Damping off, leaf spots, dieback, wilt, and fruit and root rot	Seed (seed coat)	*D. longicolla* *D. eres* *D. novem* *D. caulivora* *D. infecunda* *D. phaseolorum* *D. toxica*	*Glycine max* L. *Phaseolus vulgaris* L. *Lupinus angustifolius* L.
Gray mold (*Botrytis* spp.)	Water-soaked lesions on plant stems Necrotic spots on leaves	Seed (seed coat) Sclerotia in debris and soil	*B. cinerea* *B. fabae*	*Cicer arietinum* L. *Phaseolus vulgaris* L. *Pisum sativum* L. *Lens culinaris* Medik. *Vicia faba* L. *Pisum sativum* L.
White mold (*Sclerotinia sclerotiorum*)	Water-soaked lesions on leaves, stems, and pods Seed discoloration, alteration in shape and dimensions Germination inhibition	Seed (seed coat) Sclerotia in debris and soil	*S. sclerotiorum*	*Phaseolus vulgaris* L *Glycine max* L. *Vicia faba* L.
Aphanomyces root rot *(Aphanomyces euteiches)*	Chlorosis and necrosis on the foliage Water-soaked and gray roots	Seed Plant debris	*A. euteiches*	*Pisum sativum* L. *Glycine max* L. *Lens culinaris* Medik.

**Table 2:** List of viruses reported to have a seed transmission pathway according to the leguminous host.

Crop	Virus Species	Acronym	Genus	Family
**Alfalfa** (*Medicago sativa*)				
	*Alfalfa mosaic virus*	AMV	*Alfamovirus*	*Bromoviridae*
	*Cucumber mosaic virus*	CMV	*Cucumovirus*	*Bromoviridae*
	*Lucerne transient streak virus*	LTSV	*Sobemovirus*	*Solemoviridae*
	*Lucerne australian latent virus*	LALV	*Nepovirus*	*Secoviridae*
**Clover annual medics** (*Trifolium* spp.)				
	*Alfalfa mosaic virus*	AMV	*Alfamovirus*	*Bromoviridae*
	*Bean yellow mosaic virus*	BYMV	*Potyvirus*	*Potyviridae*
	*Clover yellow vein virus*	CYVV	*Potyvirus*	*Potyviridae*
	*Pea streak virus*	PSV	*Cucumovirus*	*Bromoviridae*
**Common bean** (*Phaseolus vulgaris*)				
	*Bean common virus*	BCMV	*Potyvirus*	*Potyviridae*
	*Bean common necrosis virus*	BCMNV	*Potyvirus*	*Potyviridae*
	*Tobacco Ringspot Virus*	TRSV	*Nepovirus*	*Secoviriadae*
	*Alfalfa mosaic virus*	AMV	*Alfamovirus*	*Bromoviridae*
	*Cucumber mosaic virus*	CMV	*Cucumovirus*	*Bromoviridae*
	*Bean yellow mosaic virus*	BYMV	*Potyvirus*	*Potyviridae*
	*Soybean Mosaic Virus*	SMV	*Potyvirus*	*Potyviridae*
	*Clover yellow vein virus*	CYVV	*Potyvirus*	*Potyviridae*
	*Cowpea chlorotic virus*	CCMV	*Bromovirus*	*Bromoviridae*
**Cowpea** (*Vigna unguiculata*)				
	*Seedborne blackeye cowpea mosaic strain*	BCMV	*Potyvirus*	*Potyviridae*
	*Cowpea aphid-borne mosaic virus*	CABMV	*Potyvirus*	*Potyviridae*
	*Cowpea mosaic virus*	CPMV	*Comovirus*	*Secoviridae*
	*Cowpea mottle virus*	CPMoV	*Gammacarmovirus*	*Tombusviridae*
	*Cucumber mosaic virus*	CMV	*Cucumovirus*	*Bromoviridae*
	*Cowpea chlorotic virus*	CCMV	*Bromovirus*	*Bromoviridae*
	*Southern bean mosaic virus*	SBMV	*Sobemovirus*	*Solemoviridae*
**Chickpea** (*Cicer aietinum*)				
	*Alfalfa mosaic virus*	AMV	*Alfamovirus*	*Bromoviridae*
	*Bean yellow virus*	BYMV	*Potyvirus*	*Potyviridae*
	*Cucumber mosaic virus*	CMV	*Cucumovirus*	*Bromoviridae*
	*Pea seedborne virus*	PSbMV	*Potyvirus*	*Potyviridae*
	*Pea enantion mosaic virus-1*	PEMV1	*Enamovirus*	*Luteoviridae*
	*Tomato mottle mosaic virus*	ToMMV	*Tobamovirus*	
**Faba bean** (*Vicia faba*)				
	*Bean yellow mosaic virus*	BYMV	*Potyvirus*	*Potyviridae*
	*Pea seedborne mosaic virus*	PSbMV	*Potyvirus*	*Potyviridae*
	*Broad bean stain virus*	BBSV		
	*Cucumber mosaic virus*	CMV	*Cucumovirus*	*Bromoviridae*
	*Pea early-browning virus*	PEBV	*Tobravirus*	*Virgaviridae*
**Groundnut** (*Arachis hypogea*)				
	*Indian peanut clump virus*	IPCV	*Pecluvirus*	*Virgaviridae*
	*Peanut clump virus*	PCV	*Pecluvirus*	*Virgaviridae*
	*Peanut mottle virus*	PeMoV	*Potyvirus*	*Potyviridae*
	*Bean common mosaic virus*	BCMV	*Potyvirus*	*Potyviridae*
	*Tobacco streak virus*	TSV	*Ilarvirus*	*Bromoviridae*
	*Cucumber mosaic virus*	CMV	*Cucumovirus*	*Bromoviridae*
**Field pea** (*Pisum sativum*)				
	*Alfalfa mosaic virus*	AMV	*Alfamovirus*	*Bromoviridae*
	*Bean yellow virus*	BYMV	*Potyvirus*	*Potyviridae*
	*Pea early-browning virus*	PEBV	*Tobravirus*	*Virgaviridae*
	*Pea enantion mosaic virus-1*	PEMV1	*Enamovirus*	*Luteoviridae*
	*Pea seedborne virus*	PSbMV	*Potyvirus*	*Potyviridae*
	*Pea streak virus*	PSV	*Cucumovirus*	*Bromoviridae*
	*Clover yellow vein virus*	CYVV	*Potyvirus*	*Potyviridae*
	*Red clover mosaic virus*	RCVMV	*Carlavirus*	*Betaflexiviridae*
**Lentil** (*Lens culinaria*)				
	*Alfalfa mosaic virus*	AMV	*Alfamovirus*	*Bromoviridae*
	*Bean yellow virus*	BYMV	*Potyvirus*	*Potyviridae*
	*Broad bean stain virus*	BBSV	*Comovirus*	*Secoviridae*
	*Cucumber mosaic virus*	CMV	*Cucumovirus*	*Bromoviridae*
	*Pea enantion mosaic virus-1*	PEMV1	*Enamovirus*	*Luteoviridae*
	*Pea streak virus*	PSV	*Cucumovirus*	*Bromoviridae*
	*Pea seedborne virus*	PSbMV	*Potyvirus*	*Potyviridae*
**Soybean** (*Glycine max*)				
	*Alfalfa mosaic virus*	AMV	*Alfamovirus*	*Bromoviridae*
	*Bean pod mottle virus*	BPMV	*Comovirus*	*Secoviriadae*
	*Soybean mosaic virus*	SMV	*Potyvirus*	*Potyviridae*
	*Tobacco ringspot virus*	TRSV	*Nepovirus*	*Secoviriadae*

**Table 3:** List of seed-transmitted viruses impacting leguminous hosts.

Virus Family or Other Group/Genus	Virus Species	Acronym	Transmission	Hosts	Rate of Seed Transmission (%) *
Family *Bromoviridae*					
*Alfamovirus*	*Alfalfa mosaic virus*	AMV	Aphids, seeds	*Medicago sativa* L. *Trifolium* spp. *Phaseolus vulgaris* L. *Vigna unguiculata* L. *Cicer arietinum* L. *Vicia faba* L. *Arachis ipogea* L. *Glycine max* L. *Lens culinaris* Medik.	0.2–74 60–70 0.7–4.9 7–82 0.1–1 0.4 - 5–9 0.1–5
*Cucumovirus*	*Cucumber mosaic virus*	CMV	Aphids, seeds	*Medicago sativa* L. *Trifolium* spp. *Phaseolus vulgaris* L. *Vigna unguiculata* L. *Cicer arietinum* L. *Vicia faba* L. *Arachis ipogea* L. *Glycine max* L. *Lens culinaris* Medik.	0.1–0.3 8–10 Up to 100 4–37 0.1–1 0–10 1.3 50–95 7–9
**Family *Potyviridae***					
*Potyvirus*	*Bean yellow mosaic virus*	BYMV	Aphids, seeds	*Trifolium* spp. *Phaseolus vulgaris* L. *Cicer arietinum* L. *Vicia faba* L. *Pisum sativum* L. *Lens culinaris* Medik.	12–15 7 3 0.1–9.8 10–30 18
	*Pea seedborne mosaic virus*	PSbMV	Aphids, seeds	*Pisum sativum* L. *Cicer arietinum* L. *Vicia faba* L. *Lens culinaris* Medik.	8–90 1 3 44
	*Soybean mosaic virus*	SMV	Aphids, seeds	*Glycine max* L.	Up to 75
	*Bean common mosaic virus*	BCMV	Aphids, seeds	*Phaseolus vulgaris* L. *Vigna unguiculata* L.	2–80 0.8–12.4

* Seed transmission rates according to Sastry, 2013 [192]; Latham and Jones, 2001 [180].

## Data Availability

The data reported in this review are publicly available in databases such as EPPO Global Database, CABI, Pubmed, FAO, ISTA methods.

## Supplementary Materials

The following supporting information can be downloaded at https://www.mdpi.com/article/10.3390/plants12102040/s1. Table S1: Categorization of seedborne pathogens of legumes in global territories.
