# Supplementary material for: Leguminous Seedborne Pathogens: Seed Health and Sustainable Crop Management
Source: Plants (Basel). 2023 May 19;12(10):2040. doi: 10.3390/plants12102040 (PMC10221191; doi:10.3390/plants12102040)
Supplement: Supplementary file 1 [file plants-12-02040-s001.zip › plants-2349835-supplementary.pdf]

Supplementary Table S1. Categorization of seedborne pathogens of legumes in global territories.

Pathogen	Country	Categorization	Year of addiction
Colletotrichum lindemuthianum	Egypt	RNQP	2018
Aphanomyces euteiches	Mexico	Quarantine pest	2018
Ascochyta fabae	Egypt	A1 list	2018
Ascochyta phaseolorum	Mexico	Quarantine pest	2018
Ascocyta pinodes	Mexico	Quarantine pest	2018
Ascochyta pinodella	China	Quarantine pest	2021
Ascochyta rabiei	Brasil	A1 list	2018
Ascochyta rabiei	Chile	A1 list	2019
Ascochyta rabiei	Mexico	Quarantine pest	2018
Botrytis cinerea	Switzerland	RNQP	2019
Botrytis cinerea	United Kingdom	RNQP	2020
Botrytis cinerea	EU	RNQP (Annex IV)	2019
Botrytis fabae	Brasil	A1 list	2018
Diaporthe longicolla	Mexico	Quarantine pest	2018
Diaporthe phaseolorum	Israel	Quarantine pest	2009
Diaporthe phaseolorum	Uzbekistan	A1 list	2008
Phoma exigua	Mexico	Quarantine pest	2018
Rhizoctonia solani	Mexico	Quarantine pest	2018
Rhizoctonia solani	Switzerland	RNQP	2019
Rhizoctonia solani	United Kingdom	RNQP	2020
Rhizoctonia solani	EU	RNQP (Annex IV)	2019
Sclerotinia sclerotiorum	Egypt	RNQP	2018
Sclerotinia sclerotiorum	Switzerland	RNQP	2019
Sclerotinia sclerotiorum	United Kingdom	RNQP	2020
Sclerotinia sclerotiorum	EU	RNQP (Annex IV)	2019
Xanthomonas axonopodis pv. *phaseoli*	Egypt	A1 list	2018
Xanthomonas axonopodis pv. *phaseoli*	Morocco	Quarantine pest	2018

Pathogen	Country	Categorization	Year of addiction
Xanthomonas axonopodis pv. *phaseoli*	Tunisia	Quarantine pest	2012
Xanthomonas axonopodis pv. *phaseoli*	Bahrain	A2 list	2003
Xanthomonas axonopodis pv. *phaseoli*	Israel	Quarantine pest	2009
Xanthomonas axonopodis pv. *phaseoli*	Jordan	A2 list	2013
Xanthomonas axonopodis pv. *phaseoli*	Azerbaijan	A1 list	2007
Xanthomonas axonopodis pv. *phaseoli*	Switzerland	RNQP	2019
Xanthomonas axonopodis pv. *phaseoli*	Türkiye	A2 list	2016
Xanthomonas axonopodis pv. *phaseoli*	United Kingdom	RNQP	2020
Xanthomonas axonopodis pv. *phaseoli*	EPPO	A2 list	1975
Xanthomonas axonopodis pv. *phaseoli*	EU	RNQP (Annex IV)	2019
Xanthomonas citri pv. *fuscans*	Switzerland	RNQP	2019
Xanthomonas citri pv. *fuscans*	United Kingdom	RNQP	2020
Xanthomonas citri pv. *fuscans*	EPPO	A2 list	2021
Xanthomonas citri pv. *fuscans*	EU	RNQP	2019
Xanthomonas citri pv. *vignicola*	Egypt	RNQP	2018
Xanthomonas citri pv. *vignicola*	Mexico	Quarantine pest	2018
Xanthomonas citri pv. *vignicola*	Jordan	A2 list	2013
Xanthomonas citri pv. *vignicola*	Switzerland	RNQP	2019

Pathogen	Country	Categorization	Year of addiction
Xanthomonas citri pv. *vignicola*	United Kingdom	RNQP	2020
Xanthomonas citri pv. *vignicola*	EU	RNQP	2019
Pseudomonas syringae pv. *glycinea*	Mexico	Quarantine pest	2018
Pseudomonas syringae pv. *phaseolicola*	Paraguay	A1 list	1995
Pseudomonas syringae pv. *phaseolicola*	Uruguay	A1 list	1995
Pseudomonas syringae pv. *phaseolicola*	Bahrain	A1 list	2003
Pseudomonas syringae pv. *phaseolicola*	China	Quarantine pest	2021
Pseudomonas syringae pv. *phaseolicola*	Israel	Quarantine pest	2009
Pseudomonas syringae pv. *phaseolicola*	Jordan	A2 list	2013
Pseudomonas syringae pv. *pisi*	Egypt	A1 list	2018
Pseudomonas syringae pv. *pisi*	Tunisia	Quarantine pest	2012
Pseudomonas syringae pv. *pisi*	Chile	A1 list	2019
Pseudomonas syringae pv. *pisi*	Paraguay	A1 list	1995
Pseudomonas syringae pv. *pisi*	China	Quarantine pest	2021
Pseudomonas syringae pv. *pisi*	Israel	Quarantine pest	2009
Pseudomonas syringae pv. *pisi*	Jordan	A2 list	2013
Pseudomonas syringae pv. *pisi*	Türkiye	A1 list	2016
Pseudomonas syringae pv. *syringae*	Egypt	RNQP	2018
Pseudomonas syringae pv. *syringae*	Mexico	Quarantine pest	2018
Pseudomonas syringae pv. *syringae*	Jordan	A2 list	2013

Pathogen	Country	Categorization	Year of addiction
Pseudomonas syringae pv. *syringae*	Switzerland	RNQP	2019
Pseudomonas syringae pv. *syringae*	United Kingdom	RNQP	2020
Pseudomonas syringae pv. *syringae*	EU	RNQP	2019
Pseudomonas viridiflava	Mexico	Quarantine pest	2018
Pseudomonas viridiflava	Switzerland	RNQP	2019
Pseudomonas viridiflava	United Kingdom	RNQP	2020
Pseudomonas viridiflava	EU	RNQP	2019
Curtobacterium flaccumfaciens pv. *flaccumfaciens*	Egypt	A1 list	2018
Curtobacterium flaccumfaciens pv. *flaccumfaciens*	Morocco	Quarantine pest	2018
Curtobacterium flaccumfaciens pv. *flaccumfaciens*	Tunisia	Quarantine pest	2012
Curtobacterium flaccumfaciens pv. *flaccumfaciens*	Argentina	A1 list	2019
Curtobacterium flaccumfaciens pv. *flaccumfaciens*	Chile	A1 list	2019
Curtobacterium flaccumfaciens pv. *flaccumfaciens*	Paraguay	A1 list	1993
Curtobacterium flaccumfaciens pv. *flaccumfaciens*	Bahrain	A1 list	2003
Curtobacterium flaccumfaciens pv. *flaccumfaciens*	China	Quarantine pest	2021
Curtobacterium flaccumfaciens pv. *flaccumfaciens*	Israel	Quarantine pest	2009
Curtobacterium flaccumfaciens pv. *flaccumfaciens*	Jordan	A1 list	2013

Pathogen	Country	Categorization	Year of addiction
Curtobacterium flaccumfaciens pv. *flaccumfaciens*	Georgia	A1 list	2018
Curtobacterium flaccumfaciens pv. *flaccumfaciens*	Switzerland	A1 list	2019
Curtobacterium flaccumfaciens pv. *flaccumfaciens*	Türkiye	A1 list	2016
Curtobacterium flaccumfaciens pv. *flaccumfaciens*	United Kingdom	A1 list	2020
Curtobacterium flaccumfaciens pv. *flaccumfaciens*	APPPC	A1 list	1993
Curtobacterium flaccumfaciens pv. *flaccumfaciens*	COSAVE	A2 list	2018
Curtobacterium flaccumfaciens pv. *flaccumfaciens*	EPPO	A2 list	1975
Curtobacterium flaccumfaciens pv. *flaccumfaciens*	EU	A1 Quarantine pest	2019
Curtobacterium flaccumfaciens pv. *flaccumfaciens*	IAPSC	A1 list	1989
Alfalfa mosaic virus	Egypt	RNQP	2018
Alfalfa mosaic virus	Bahrain	A2 list	2003
Alfalfa mosaic virus	Jordan	A2 list	2013
Bean yellow mosaic virus	Egypt	RNQP	2018
Bean yellow mosaic virus	Bahrain	A2 list	2003
Bean yellow mosaic virus	Jordan	A2 list	2013
Cucumber mosaic virus	Egypt	RNQP	2018
Cucumber mosaic virus	Bahrain	A2 list	2003
Cucumber mosaic virus	Jordan	A2 list	2013
Cucumber mosaic virus	Switzerland	RNQP	2019
Cucumber mosaic virus	United Kingdom	RNQP	2020

Pathogen	Country	Categorization	Year of addiction
Cucumber mosaic virus	EU	RNQP	2019
Cucumber mosaic virus	IAPSC	A2 list	1992
Pea seed-borne mosaic virus	Egypt	A2 list	2018
Pea seed-borne mosaic virus	Argentina	A1 list	2019
Pea seed-borne mosaic virus	Mexico	Quarantine pest	2018
Pea seed-borne mosaic virus	Bahrain	A1 list	2003
Pea seed-borne mosaic virus	Jordan	A2 list	2013
Bean common mosaic virus	Egypt	A2 list	2018
Bean common mosaic virus	Morocco	Quarantine pest	2018
Bean common mosaic virus	Argentina	A1 list	2019
Bean common mosaic virus	United States of America	Quarantine pest	2000
Bean common mosaic virus	Bahrain	A1 list	2003
Bean common mosaic virus	Jordan	A2 list	2013
Soybean mosaic virus	Egypt	A2 list	2018
Lucerne australian latent virus	United States of America	Quarantine pest	2000
Lucerne australian latent virus	Jordan	A1 list	2013
Clover yellow vein virus	Brasil	A1 list	2018
Tobacco ringspot virus	Egypt	A2 list	2018
Tobacco ringspot virus	Morocco	Quarantine pest	2018
Tobacco ringspot virus	Tunisia	Quarantine pest	2012
Tobacco ringspot virus	Argentina	A1 list	2019
Tobacco ringspot virus	Brazil	A1 list	2018
Tobacco ringspot virus	Canada	Quarantine pest	2019

Pathogen	Country	Categorization	Year of addiction
Tobacco ringspot virus	Mexico	Quarantine pest	2018
Tobacco ringspot virus	Paraguay	A1 list	1995
Tobacco ringspot virus	China	Quarantine pest	2021
Tobacco ringspot virus	Israel	Quarantine pest	2009
Tobacco ringspot virus	Jordan	A2 list	2013
Tobacco ringspot virus	Kazakhstan	A1 list	2017
Tobacco ringspot virus	Moldova	Quarantine pest	2017
Tobacco ringspot virus	Norway	Quarantine pest	2012
Tobacco ringspot virus	Russia	A2 list	2014
Tobacco ringspot virus	Switzerland	A1 list	2019
Tobacco ringspot virus	Türkiye	A1 list	2016
Tobacco ringspot virus	Ukraine	A1 list	2019
Tobacco ringspot virus	United Kingdom	A1 list	2020
Tobacco ringspot virus	EPPO	A2 list	1995
Tobacco ringspot virus	EU	A1 Quarantine pest	2019
Southern bean mosaic virus	Argentina	A1 list	2019
Southern bean mosaic virus	Paraguay	A1 list	1995
Southern bean mosaic virus	China	Quarantine pest	2021
Southern bean mosaic virus	Jordan	A1 list	2013
Tomato mottle mosaic virus	EPPO	Alert list	2020
Broad bean stain virus	Egypt	RNQP	2018
Broad bean stain virus	China	Quarantine pest	2021
Broad bean stain virus	Jordan	A2 list	2013

Pathogen	Country	Categorization	Year of addiction
Indian peanut clump virus	United States of America	Quarantine pest	2000
Peanut clump virus	United States of America	Quarantine pest	2000
Peanut clump virus	Bahrain	A1 list	2003
Tobacco streak virus	Mexico	Quarantine pest	2018
Tobacco streak virus	Paraguay	A1 list	1995
Tobacco streak virus	Israel	Quarantine pest	2009
Tobacco streak virus	Jordan	A1 list	2013
Tobacco streak virus	Türkiye	A1 list	2016
Bean pod mottle virus	Argentina	A1 list	2019
Bean pod mottle virus	Bahrain	A1 list	2003
Bean pod mottle virus	China	Quarantine pest	2021

Data retrieved from EPPO Global Database accessed on April, 28th 2023.
